# Edible Natural Deep Eutectic Solvents (NADESs)-Based Polyphenolic Extracts: An Eco-Sustainable Alternative for Grape Pomace Valorization

**DOI:** 10.3390/molecules31101665

**Published:** 2026-05-15

**Authors:** Vincenzo Roselli, Rosalba Leuci, Marco Paparella, Gianluca Pugliese, Carlos Luz, Alessandra Cimbalo, Lara Manyes, Luigi Tarricone, Vincenzo Tufarelli, Lucia Gambacorta, Giuseppe Meca, Luca Piemontese

**Affiliations:** 1 Department of Pharmacy-Pharmaceutical Sciences, University of Bari Aldo Moro, Campus E. Quagliariello, Via E. Orabona, 4, 70126 Bari, BA, Italy; 2 Department Food Science and Toxicology, Faculty of Pharmacy, University of Valencia, Ave. Vicent Andrés Estellés 22, 46100 Burjassot, Spain; 3 Institute of Science of Food Production (ISPA), Research National Council (CNR), Via Amendola, 122/O, 70126 Bari, BA, Italy; 4 Department of Precision and Regenerative Medicine and Jonian Area (DiMePRe-J), Section of Veterinary Science and Animal Production, University of Bari Aldo Moro, 70010 Valenzano, BA, Italy; 5 Research Centre for Viticulture and Enology, Council for Agricultural Research and Agricultural Economy Analysis (CREA-VE), Via Casamassima, 148, 70010 Turi, BA, Italy

**Keywords:** NADESs, grape pomace, bioactive molecules, HPLC-DAD, metabolomics

## Abstract

Reusing waste materials is a sustainable practice to reduce the impact of human activities on the global ecosystem. In particular, agro-industrial waste can be explored as an innovative and green source of beneficial bioactive compounds. For this purpose, seventeen different varieties of wine grape pomace were collected from the Apulia region (Italy) to perform extractions and qualitative–quantitative phenolic profile characterization. To ensure an environmentally friendly extraction process, natural deep eutectic solvents (NADESs) were used as privileged media. After an initial screening, *Merlot* (MEA) and *Sangiovese* (SGA) varieties were then selected for extraction with betaine/lactic acid 1:4 (mol/mol) +40% water (*w*/*w*) and choline chloride/ascorbic acid 2:1 (mol/mol) +40% water (*w*/*w*). They were further investigated by LC-QTOF-MS for a comprehensive metabolomic evaluation. Betaine-based extracts from both cultivars exhibited higher polyphenol contents than choline chloride-based ones: 175.0 and 161.8 mg/kg compared to 59.6 and 40.4 mg/kg. Qualitative antimicrobial assays confirmed the effectiveness of betaine/lactic acid (MEA) and choline chloride/ascorbic acid (SGA) extracts on *B. allii* and *E. coli*, respectively, as well as the NADES themselves, even though it is difficult to discriminate the individual effects. Finally, the evaluation of both antioxidant activity and total polyphenol content led to good results for betaine/lactic acid (2.8 and 3.2 mg TEAC/g DW and 0.92 and 0.93 mg GAE/g DW for SGA and MEA, respectively), while the content of ascorbic acid, used as a component of NADES, substantially influenced the relevant obtained results in choline chloride/ascorbic acid samples. These findings support the potential of combining NADES with polyphenols recovered from grape pomace as a promising approach for further optimization and application-oriented investigation.

## 1. Introduction

In 2015, all United Nations member states adopted the “2030 Agenda for Sustainable Development”, summarized by the seventeen Sustainable Development Goals, which established common ambitious goals for the improvement of health, education and economic growth, all while tracking and preserving the global ecosystem [1]. This provides a concrete step forward for the prosperity of people and the planet, whose ecosystem stability is threatened by intensive industrial activities. Global food production has a strong impact on the environment, and it is expected to increase by 70% to meet growing population demands by 2050 [2]. Currently, approximately 733 million people are facing hunger, while 2.33 billion are food insecure [3]. Moreover, agro-industrial waste is one of the major contributors to global pollution, with agricultural-based industries producing approximately 1.3 billion tons annually [4].

Considering agriculture-derived by-products as an innovative secondary raw material rather than waste is important to bring out their hidden potential as completely sustainable sources of bioactive molecules with a positive impact on human health [5,6,7]. Among them, phenolic compounds, which are secondary metabolites of plants, are involved in the prevention of various oxidative stress-linked diseases: neurodegenerative and cardiovascular disorders, cancer, and diabetes [8,9]. Moreover, in addition to their strong antioxidant activity, they exhibit broad-spectrum efficacy against different pathogens, including viruses, bacteria and fungi [9,10]. Based on molecular structure, polyphenols can be classified as phenolic acids, flavonoids, tannins, stilbenes, coumarins or lignans [5,8,9].

Grapes are one of the most widespread fruit crops, with 7.2 million hectares of land used for vine cultivation. Roughly 52% of produced grapes are used as a starting material by the winemaking industry [11], with up to 258 million hectoliters of wine produced in 2023 [9,12]. Grape pomace is the main solid by-product of this industry, accounting for approximately for 30% *w*/*w* of the total starting material [13,14]. The richness of this secondary material in phenolic compounds [15,16] has already led to several studies exploring different extraction techniques, with a focus on green approaches [17,18].

Deep eutectic solvents (DESs) have already been proposed as green extracting phases in substitution for the conventional ones [9]. Their low volatility, toxicity, and flammability, along with high tunability and thermal and chemical stability, make DESs safe and versatile alternatives to conventional organic solvents, which are often effective but unsafe and polluting. [9,19]. Prevention of waste, designing of safer and effective chemicals, use of renewable material and minimization of chemical accidents, such as explosions and releases, are the main points of the well-known Twelve Principles of Green Chemistry of Paul Anastas and John Warner, with which the use of DESs is well aligned [20].

DES formation is a sustainable process, requiring little time and energy expenditure. It occurs through the formation of hydrogen bonds between two or more liquid or solid constituents mixed in specific proportions, of which at least one acts as a hydrogen-bonding acceptor (HBA) and one as a hydrogen-bonding donor (HBD) [9,21]. The resulting solvent shows a significant reduction, compared to the starting compounds, of the melting point [9,21]. Natural deep eutectic solvents (NADESs) are a subcategory of DESs, in which the starting constituents are natural molecules, such as organic acids, sugars and amino acids, present in living cells and abundant in daily food [9,21,22]. For this reason, they are usually even cheaper, more sustainable and safer than conventional DESs [23]. Moreover, the high solubility of metabolites and even macromolecules in NADESs [23,24] makes them an exciting tool for the sustainable extraction of valuable bioactive molecules.

This work investigated the existing gap between the already widely studied and discussed use of DESs/NADESs to recover bioactive molecules from grape pomace [25,26,27,28] and a more practical application of these extracts for human and/or animal nutrition through the evaluation of their chemical and biological activity. Additionally, only cultivars of grape pomace from the Apulia region (Italy) were considered in order to focus our efforts on a region with a strong agro-industrial background in the Mediterranean area.

In the present study, three previously selected NADESs [9] were used to perform solid–liquid extractions of phenolic compounds from grape pomace. The extracts were characterized through High Performance Liquid Chromatography (HPLC) coupled with a UV-Diode Array Detector (UV-DAD), and the most interesting samples were further chemically investigated by LC-ESI-QTOF-MS. Finally, antioxidant activity, total polyphenol content and qualitative antimicrobial activity were evaluated to assess the potential of these extracts as ready-to-use food and/or feed supplements.

## 2. Materials and Methods

### 2.1. Chemicals and Reagents

Milli-Q water (resistivity < 18 Ωcm) was provided through a Milli-Q purification system (Millipore, Billerica, MA, USA). Acetonitrile (ACN), methanol (MeOH), ethanol (EtOH), formic acid (FA), choline chloride (ChCl), betaine hydrochloride (Bet), ascorbic acid (AA), lactic acid (LA), urea, and Folin–Ciocalteu reagent were purchased from Merck (Darmstadt, Germany). Phosphate buffer saline (PBS) was supplied by Thermo Fisher Scientific (Waltham, MA, USA). 2,2′-azino-bis(3-ethylbenzothiazoline-6-sulfonic acid) diammonium salt (ABTS), 6-hydroxy-2,5,7,8-tetramethylchroman-2-carboxylic acid (Trolox), and phenolic compounds’ standards (gallic acid, syringic acid, coumaric acid, caffeic acid, ferulic acid, chlorogenic acid, resveratrol, kaempferol 3-O-galactoside, rutin, quercetin and catechin) were provided from common suppliers; they were of analytical grade and used without further purification. The polyphenols were of purity suitable for use as standards.

### 2.2. Collecting and Sampling of Grape Pomace

Seventeen red and white grape pomaces (GPs) (Table 1) were collected during the 2024 vintage from different parts of Apulia (Italy). This is one of the main winemaking regions of Italy, with over 1.4 million hectoliters of wine produced from more than 90,000 hectares of vineyards in 2024 (ISTAT, 2024) [29]. All the samples investigated in this study were obtained immediately after the winemaking process. Moreover, for a complete characterization of this by-product, grape cultivation treatment (conventional, organic, biodynamic) was taken into account. Organic farming could represent a further interesting sustainable choice to limit environmental impact in agricultural production and is usually connected to the production of niche, high-quality wines [30]. All grape pomaces were dried in a drying oven (Mod FED 720, BINDER GmbH, Tuttlingen, Germany) at 40 °C for 48–72 h.

### 2.3. Proximate Composition Analysis of GP

As a first step, the basic chemical composition of the different GP cultivars was determined by evaluating their main nutritional and structural components. More specifically, the following parameters were analyzed: dry matter, crude protein (CP), crude fiber (CF), crude lipids, and total ash content. Dry matter content (DM, method 945.15) was determined by a moisture analyzer (35 °C for 72 h) [31]. Crude protein (CP) content was assessed using a Bherotest Steam distillation apparatus, fully automatic, S5 (Behr Labor-Technik, Düsseldorf, Germany), equipped with an external titrator TitroLine^®^ 5000 (SI Analytics, Xylem Inc., Washington, DC, USA) according to the Kjeldahl method (990.03). The CP content was then obtained by multiplying total nitrogen × 6.25 [31]. For total CF content, an automated system for precise measurement of fiber fractions, Ankom 220 Fiber Analyzer (ANKOM Technology, Macedon, NY, USA) was employed following the method of Van Soest et al. [32]. Crude lipids were determined through a petroleum ether-based extraction of the fats from the samples (method 922.06) [33] using a Soxhlet apparatus (LAUDA, Lauda-Königshofen, Germany). Finally, for total ash content, 3 g of representative samples were weighed and placed in a muffle furnace (Mod ELF 1100, Carbolite Gero Ltd., Hope, Derbyshire, UK) for 4 h at 550 °C (method 967.05) [31]. All chemical analyses were conducted in triplicate.

### 2.4. Preparation of NADESs for Extraction Procedures

The preparation of all NADESs needed for the extractions was carried out under controlled stirring and temperature conditions. Extraction procedures included orbital shaking and centrifugation steps.

Three NADESs (Table 2) were prepared using ChCl and Bet as HBAs and urea, LA and AA as HBDs. For all NADEs, 40% of water content, calculated on the total weight of the solvent prepared, was used to minimize viscosity without significantly affecting the extraction of polar molecules [34].

Choline chloride/ascorbic acid 2:1 (mol/mol + 40% H_2_O; ChCl/AA) NADES was prepared according to the method described by Liu W. et al. [35] with modifications. Before mixing the two components, they were dissolved in a sufficient quantity of water to form two solutions (room temperature, 20–30 min). Then they were merged, and the excess of water was removed by rotary evaporator (Heidolph, Schwabach, Germany) (40 °C, 50 mbar, 60–120 min). A similar procedure, with different rotary evaporator parameters (55 °C, 80 mbar, 60–120 min), was followed to prepare betaine hydrochloride/lactic acid 1:4 (mol/mol + 40% H_2_O; Bet/LA) NADES. This preparation method was optimized starting from that of Liu Y. et al. [36]. Finally, choline chloride/urea 1:2 (mol/mol + 40% H_2_O; ChCl/urea) NADES was obtained by mixing and stirring the corresponding weighed components and, after a completely homogenous solution was obtained, the calculated amount of water was added [37]. An ethanol/water 70% (wt/wt) solution was prepared as a reference solvent in the extractions [38,39,40].

### 2.5. Extraction of Phenolic Compounds from Real Samples

Seventeen grape pomace samples were initially collected and subjected to HPLC-DAD screening. Based on their phenolic profiles, a subset of samples was selected for further LC-QTOF-MS analysis. All extractions were performed in duplicate (*n* = 2 independent extractions) on both fresh and dried GPs. A homogenous sample of GP was weighed (5 g) and mixed with the solvent according to a 1:4 ratio (wt/wt). The experiment, as similarly described in previous studies involving DESs or VOCs as extraction media [9,41], lasts 1 h under shaking conditions (250 rpm, 25 °C) in closed jars. Then, after excluding the solid part through decantation and centrifugation (4000 rpm, 25 °C, 15 min), a total of five samples were obtained from each extract and represent analytical aliquots used for HPLC-DAD or LC-QTOF-MS. Both fresh and dried grape pomace samples were analyzed by HPLC-DAD, while LC-QTOF-MS analysis was performed only on selected samples. All extractions were performed in duplicate (*n* = 2 independent extractions).

### 2.6. HPLC-DAD Analysis

HPLC-DAD analysis was performed on all extracts, from both fresh and dried GPs, using an Agilent 1100 liquid chromatography system with a binary pump, an auto sampler with a 100 μL loop and a Zorbax SB-C18 column 5 μm (4.6 × 150 mm) (Agilent Technologies Inc., Wilmington, DE, USA). The mobile phase, whose flow rate was fixed at 0.7 mL/min (25 °C), consisted of a gradient of acidic H_2_O (with 5% of acetic acid content) (A) and MeOH (B). The gradient was set as follows: 0 min, 2% B; 35 min, 40% B; 40 min, 40% B; 55 min, 63% B; 62 min, 63% B; 66 min, 100% B; 67 min, 100% B; 75 min, 2% B; 80 min, 2% B. A spectrophotometric diode array detector (DAD) was coupled with software for Microsoft Windows 7 (OpenLAB, CSB, ChemStation Edition) for data analysis.

Before the analyses, the samples were diluted with water (1:1 *v*/*v*), centrifuged (14,500 rpm, 30 °C, 2 min), and filtered with 0.45 μm regenerated cellulose filters. An amount of 20 μL of the sample was injected (25 °C) for each run. Five different wavelengths were chosen for the DAD’s detection: 260, 280, 325, 360 and 560 nm. Analytical measurements were conducted in duplicate as technical replicates.

### 2.7. Validation Parameters for Quantification of Phenolic Compounds

For the validation of the method, commercial standards of the phenolic compounds were used to build calibration curves of each compound within a linear range of 0.008–132.5 μg/mL. Regression coefficients above 0.99 were reported. For each phenolic compound detected, the limit of detection (LOD) and limit of quantification (LOQ) were already reported in a previous investigation [9].

### 2.8. Characterization of the Metabolomic Profile by LC-QTOF-MS

LC-QTOF-MS characterization was carried out on selected dried GP cultivars (MEA and SGA), extracted with Bet/LA, ChCl/AA and ethanol 70% as reference. The analyses were performed in duplicate as technical replicates to assess instrumental repeatability, following the method described by Conti Taguali et al. [42]. Chromatographic determination was conducted using a C18 column with a binary solvent system: Milli-Q water (A) and acetonitrile (B), both acidified with 0.1% of formic acid. The following elution gradient was adopted: 0 min, 2% B; 22 min, 95% B; 25 min, 5% B. An amount of 5 μL of each sample was injected, and the flow rate was set at 0.4 mL/min. Before injecting, as for HPLC-DAD analysis, a dilution step (1:1 *v*/*v*), along with centrifugation (14,500 rpm, 30 °C, 5 min) and filtration with 0.22 μm filters, was done.

QTOF-MS (6540 Agilent Ultra High-Definition Accurate Mass), equipped with an Agilent Dual Jet Stream electrospray ionization (Dual AJS ESI) interface (in both positive and negative ionization modes), was used for mass spectrometry (MS) analysis. The operating conditions of the mass spectrometer were: gas flow: 10 L/min; gas temperature: 325 °C; nebulizer pressure: 40 psig; sheath gas flow: 12 L/min; sheath gas temperature: 295 °C; Fragmentor: 120 V; capillary voltage: 4000 V; nozzle voltage: 500 V; skimmer: 70 V; product ion scan range: 100–1500 Da; MS scan rate: 5 spectra/s; MS/MS scan rate: 3 spectra/s; collision energy: 10, 20, 40 eV; maximum pre-cursors per cycle: 2.

Data processing for metabolite identification was conducted using MassHunter Qualitative Analysis Software (version B.08.00), whereas the Personal Compound Database and Library (PCDL) was employed for compounds annotation. A library of ninety-nine compounds, built according to the most common phenolic compounds found in grape pomace through mass spectrometry, was used [43,44,45]. Metabolite identification was considered tentative, based on accurate mass and database matching. A semi-quantitative approach [46] was used to quantify all phenolic metabolites identified. A gallic acid standard curve (0.016–10 mg/L) was built, and the values obtained, expressed as mg Gallic Acid Equivalents (GAE) per kg of dry sample (DW), can be interpreted as semi-quantitative estimates, rather than the result of an absolute quantification. They enable a semi-quantitative comparison, within a wide spectrum of targeted bioactive molecules, between all the extracting solvents considered. A score threshold of 70% and a Delta mass error tolerance of 1 ppm were applied as confidence criteria.

### 2.9. Antimicrobial Activity Assays

For a more comprehensive evaluation of the extracts analyzed by LC-QTOF-MS (dried MEA and SGA grape pomaces extracted with Bet/LA, ChCl/AA and ethanol 70%), the qualitative in vitro antimicrobial activity was determined by agar diffusion test. As the focus of this assay was on performing a comparative assessment of the sample, a qualitative approach was preferred rather than a quantitative one. The experiment was performed using pathogenic fungi and bacteria, as described by Luz et al. [47]. The species used were sourced from the Colección Española de Cultivos Tipo (CECT), Valencia University, Spain and from ITEM Collection from Istituto di Scienze delle Produzioni Alimentari (ISPA): *Fusarium verticillioides* (CECT 20926), *Aspergillus flavus* (ISPA 8111), *Alternaria alternata* (CECT 2662), *Botrytis cinerea* (CECT 2100), *Botrytis allii* (CECT 2851), as pathogenic fungi which are most likely to contaminate food [48,49] and *Staphylococcus aureus* (CECT 240), *Escherichia Coli* (CECT 4782), *Listeria monocytogenes* (CECT 935) and *Salmonella enterica* (CECT 554), as pathogenic bacteria [50]. All microorganisms were obtained from reference cultures (10^8^ CFU/mL) and maintained in PDA medium or nutrient agar.

All extracts tested were initially freeze-dried. A dilution step (1:10 wt/wt) was performed for NADES’ extracts before removing all the water using a Labconco FreeZone 2.5 L −50 °C Benchtop Freeze Dryer System (Labconco Corporation, Kansas City, MO, USA). The freeze-drying procedure was split into two parts, each of which lasted 24–48 h; between each part, the samples were stored at −80 °C. For the ethanolic extracts, the rotary evaporator was used to remove ethanol, and the freeze dryer was finally employed to eliminate the residual water. The samples tested were prepared by dissolving the powder obtained from each extract in sterile water (400 g/L). The superficial seeding of each microbial suspension was done on plates of PDA (fungi) and NBA (bacteria) medium, on which 100 μL of each sample was added. The plates were incubated at 25 °C for 72 h, and the inhibition zones formed were measured in order to estimate, qualitatively, the antimicrobial activity based on the diameter of these inhibition zones. The results of the assay, collected in triplicate, were expressed according to an empirical scale, as reported by Riolo et al. [51] with modifications: (−) no inhibition halo, (+) mean diameter inhibition halo < 8 mm, (++) mean diameter inhibition halo 8–12 mm, (+++) mean diameter inhibition halo > 12 mm.

### 2.10. Antioxidant Activity

The characterization of the samples (ethanol 70%, Bet/LA and ChCl/AA-based extracts from dried MEA and SGA) was further investigated by the detection of antioxidant activity, performed with ABTS radical cation decolorization assay, following the procedure applied by Gallego et al. [52], with modifications. An amount of 0.0114 g of ABTS powder was dissolved in 3 mL of potassium persulfate 2.45 mM solution in phosphate buffer saline (PBS) in order to obtain ABTS 7 mM radical cation stock solution. After incubation overnight, in dark conditions, this solution was diluted with PBS (pH 7.4) to have a final absorbance of 0.7 at 734 nm. An amount of 20 μL of diluted sample was mixed with 1980 μL of ABTS solution, and the resulting absorbance was measured after 6 min of incubation in dark conditions at 734 nm. The results were expressed as mg Trolox Equivalent Antioxidant Capacity (TEAC) per gram of sample in dry weight (DW) by building a standard curve (0.05–2 mM). PBS was used as a blank, and all the analysis, as well as the detection of the absorbance, were performed in duplicate.

### 2.11. Total Polyphenol Content

The determination of total polyphenol content was conducted on the same samples analyzed by the ABTS test. The experiment was performed with the Folin–Ciocalteu assay, following the method described by Alessandroni et al. [53] with modifications. An amount of 130 μL of the sample was mixed with 780 μL of distilled water. Folin–Ciocalteu 2 M solution was diluted with distilled water (1:10 *v*/*v*) and used to perform the experiment. An amount of 130 μL of this solution, along with 130 μL of Na_2_CO_3_ water solution (20%, *v*/*v*), was added to the diluted samples. The absorbance was measured at 765 nm after 1 h of incubation in dark conditions. The results were expressed as mg Gallic Acid Equivalents (GAE) per gram of sample in dry weight (DW) by building a gallic acid standard curve (140-0 mg/L). The assay was conducted in triplicate.

### 2.12. Statistical Analysis

For clarity and relevance, the most abundant phenolic compounds detected by HPLC-DAD were considered in the analysis. Specifically, catechin, quercetin, and rutin were identified as the major compounds across both fresh and dried grape pomace extracts. Results are presented as mean ± standard deviation (SD), and observed differences are described qualitatively.

Moreover, heatmaps were generated to visually represent the semi-quantitative distribution of phenolic compounds across the different samples.

Technical duplicates (instrumental injections) were performed for each extract to ensure analytical repeatability.

## 3. Results and Discussion

### 3.1. Proximate Composition Analysis of GP

The chemical composition of each GP cultivar is described in Table 3. The values found are compatible with the ones already reported for wide varieties of grape pomace [54,55]. Overall, the varieties had a moisture content which widely ranged from 35.36% to 68.46%, with *Negro Amaro* cultivar (conventional and biodynamic) that resulted to have the highest percentages. *Moscato* variety had the lowest value of CP (7.22%) and CF (12.53%) content (which was more than three times lower than the one found for *Merlot*), while *Trebbiano* had the highest value for CP (13.94%) and the lowest one for lipids (2.32%) and ash (5.3%). The MEA, MIR and PRA were found to be the most lipid-enriched GPs’ varieties.

### 3.2. Quantification of Phenolic Compounds in GPs

HPLC-DAD analysis was conducted to perform an initial screening of all studied grape pomaces. The analysis was also conducted after the drying process, since the water content of each cultivar, soon after the winemaking process, differs.

**Figure 1:**
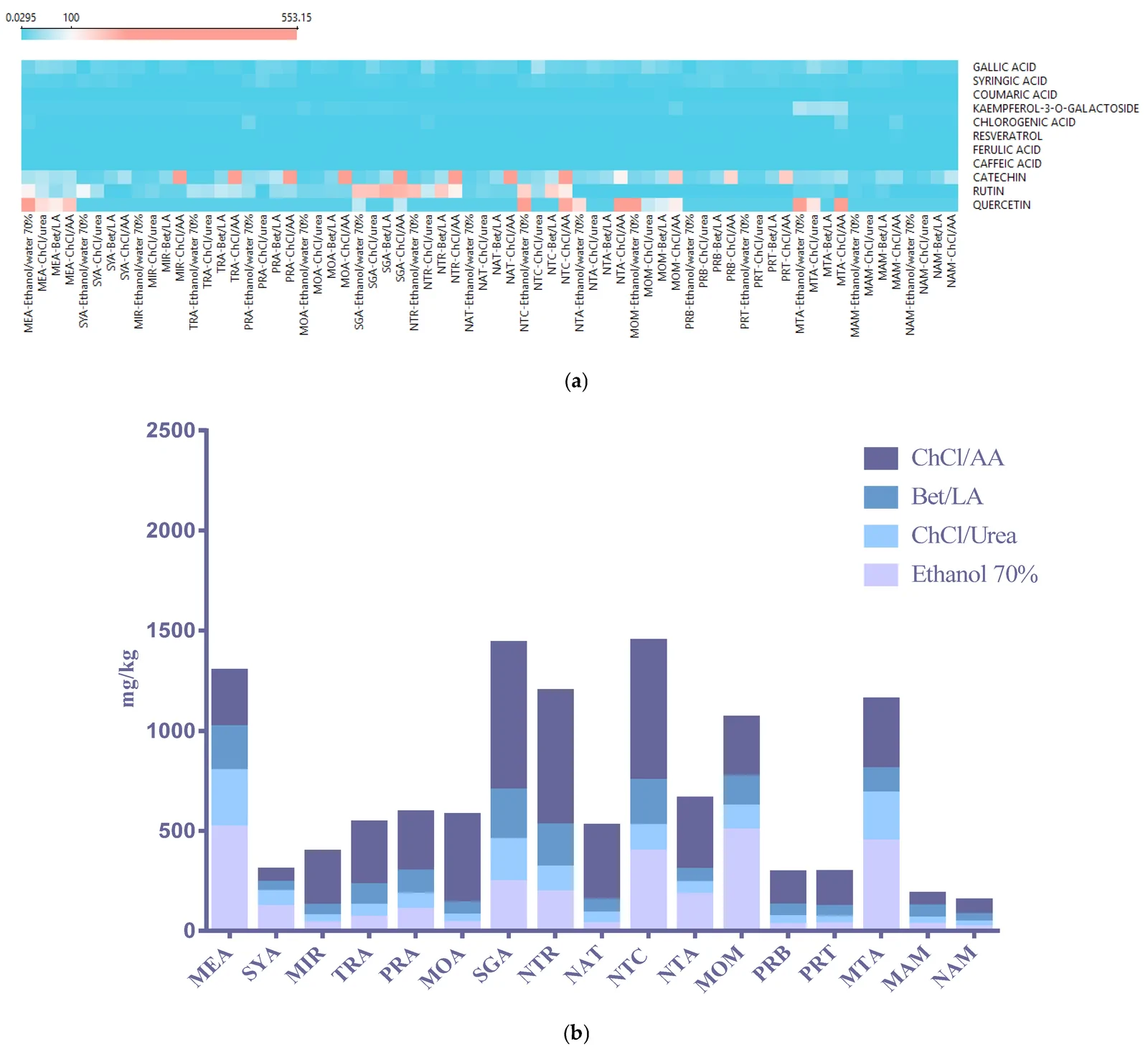
(**a**). Heatmap showing the levels of phenolic compounds (mg/kg DW) in all fresh grape pomace (GP) samples analyzed by HPLC-DAD. Each row represents a phenolic compound, and each column represents a GP extract. (**b**) Bar chart showing the total phenolic content (mg/kg DW) of fresh grape pomace extracts obtained with the four different solvents, as determined by HPLC-DAD analysis. Differences among samples are presented as qualitative trends due to the limited number of replicates.

**Figure 2:**
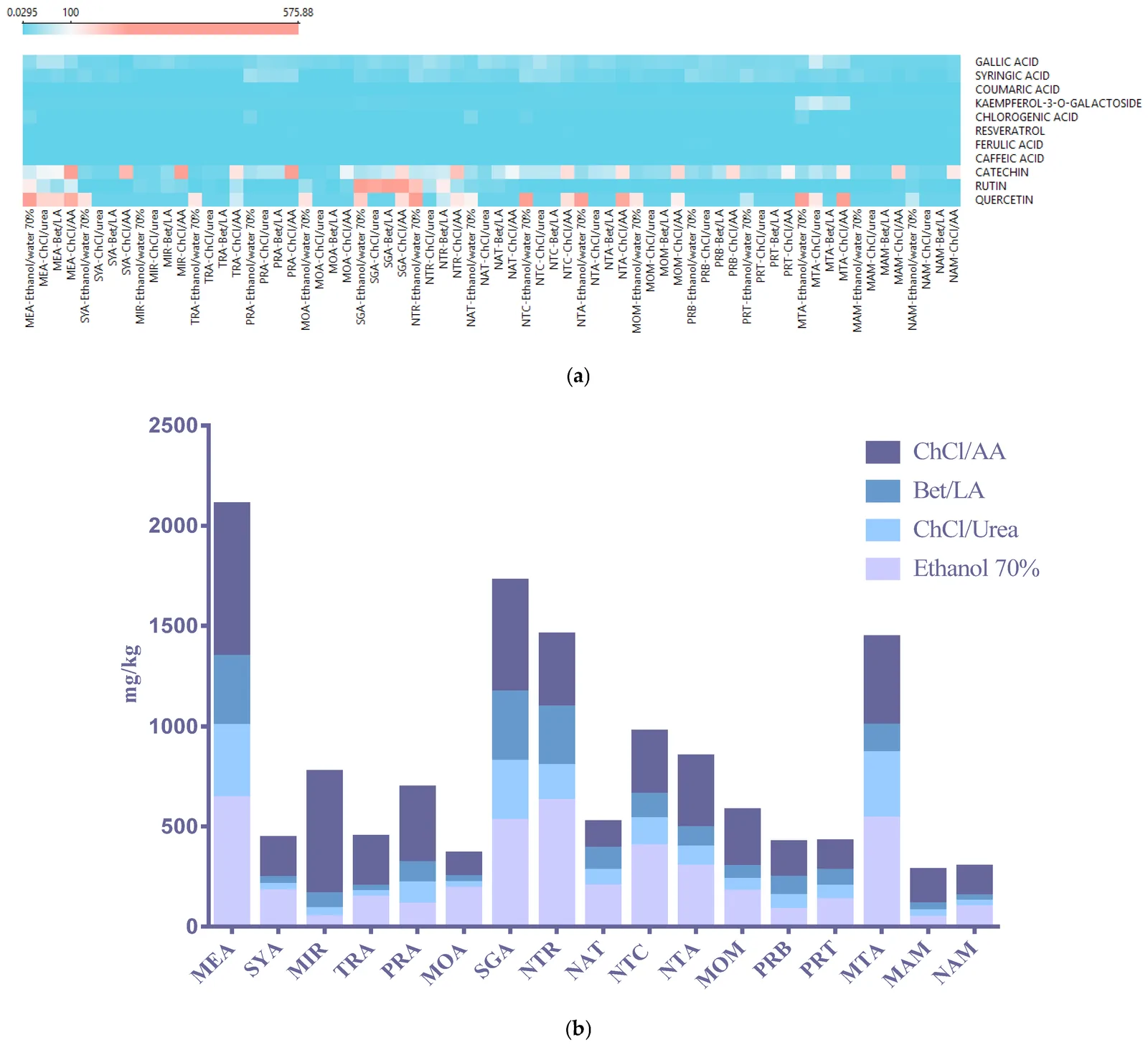
(**a**). Heatmap showing the levels of phenolic compounds (mg/kg DW) in all dried grape pomace (GP) samples analyzed by HPLC-DAD. Each row represents a phenolic compound, and each column represents a GP extract. (**b**) Bar chart showing the total phenolic content (mg/kg DW) of dried grape pomace extracts obtained with the four different solvents, as determined by HPLC-DAD analysis. Differences among samples are presented as qualitative trends due to the limited number of replicates.

All the phenolic compounds’ standards detected were reported as mg of polyphenols per kg of sample in dry weight (DW) (Tables S1 and S2 and Figure 1a,b and Figure 2a,b).

Quercetin, catechin and rutin were the most abundant polyphenols found in the samples (Figure 1a and Figure 2a), as reported also by several studies about grape pomace polyphenol composition [56,57,58,59]. In fresh GPs (Figure 1a,b), differences among solvents and cultivars were observed for quercetin recovery (Figure S3). Catechin recovery appeared to be influenced primarily by the solvent used (Figure S1) while rutin recovery varied across cultivars (Figure S2). ChCl/AA NADES was found to be the best extracting solvent for thirteen fresh GPs out of seventeen, even considering ethanol 70%, used as a reference. Moreover, aside from ethanol 70%, Bet/LA was found to be the second most effective NADES in fourteen fresh GPs out of seventeen.

NTC, SGA, MEA, NTR, MTA, and MOM were the GPs with the highest phenolic content, with total yields exceeding 1000 mg/kg DW when all the extraction solvents were considered together. Ethanol 70% was confirmed to be the best extracting solvent for *Merlot* (MEA) and *Montepulciano* (both MTA and MOM) GPs, and, in both cases, the majority (mg/kg DW) of all phenolic compounds detected was constituted by quercetin. For the other three cultivars, the reference solvent was surpassed by ChCl/AA NADES, which provided extracts more abundant in catechin and rutin. On the other hand, the poorest fresh GPs were MAM and NAM, with yields lower than 200 mg/kg DW, calculated as the sum of the polyphenols extracted using all four solvents investigated. For *Malbec* GP (MAM), Bet/LA and ChCl/AA NADESs had very similar yields (around 60 mg/kg DW), and for NAM, ChCl/AA clearly surpassed all the other solvents. In both cultivars, the major bioactive molecule detected, for each solvent used, was catechin. Considering the samples referred to dried GPs (Figure 2a,b), differences in solvent and cultivar were both relevant for quercetin recovery (Figure S6); variations in catechin and rutin recovery (Figures S4 and S5) were observed depending on the solvent and cultivar.

ChCl/AA NADES generally led to higher polyphenol yields compared to other solvents, recovering more polyphenols than all the other three solvents in twelve cases out of seventeen and being the best NADES in all cases. Bet/LA showed similar extraction yields to ChCl/urea NADES, and in several cases, higher yields were observed; moreover, in eleven cases out of seventeen, Bet/LA was better than ChCl/urea NADES. Even in this case, MAM and NAM were confirmed to be the worst dried GPs for polyphenol recovery. In both cultivars, ChCl/AA NADES was the best solvent, with catechin accounting for more than 80% of the total polyphenol yield. Even the profile of the best dried GPs was maintained: MEA, SGA, NTR, MTA, NTC. Each of these cultivars had total yields from around 1000 mg/kg DW up to more than 2000 mg/kg DW when considering the sum of the yields of all four solvents tested. In *Merlot* (MEA) and *Sangiovese* (SGA) GPs, ChCl/AA NADES reached better yields than ethanol 70% (761.5 compared to 637 mg/kg DW and 555.8 compared to 523.3 mg/kg DW respectively) with a major content in quercetin and rutin respectively. The other cultivars had, as the best extracting solvents, ethanol 70%, in which quercetin was the most abundant bioactive compound.

**Figure 3:**
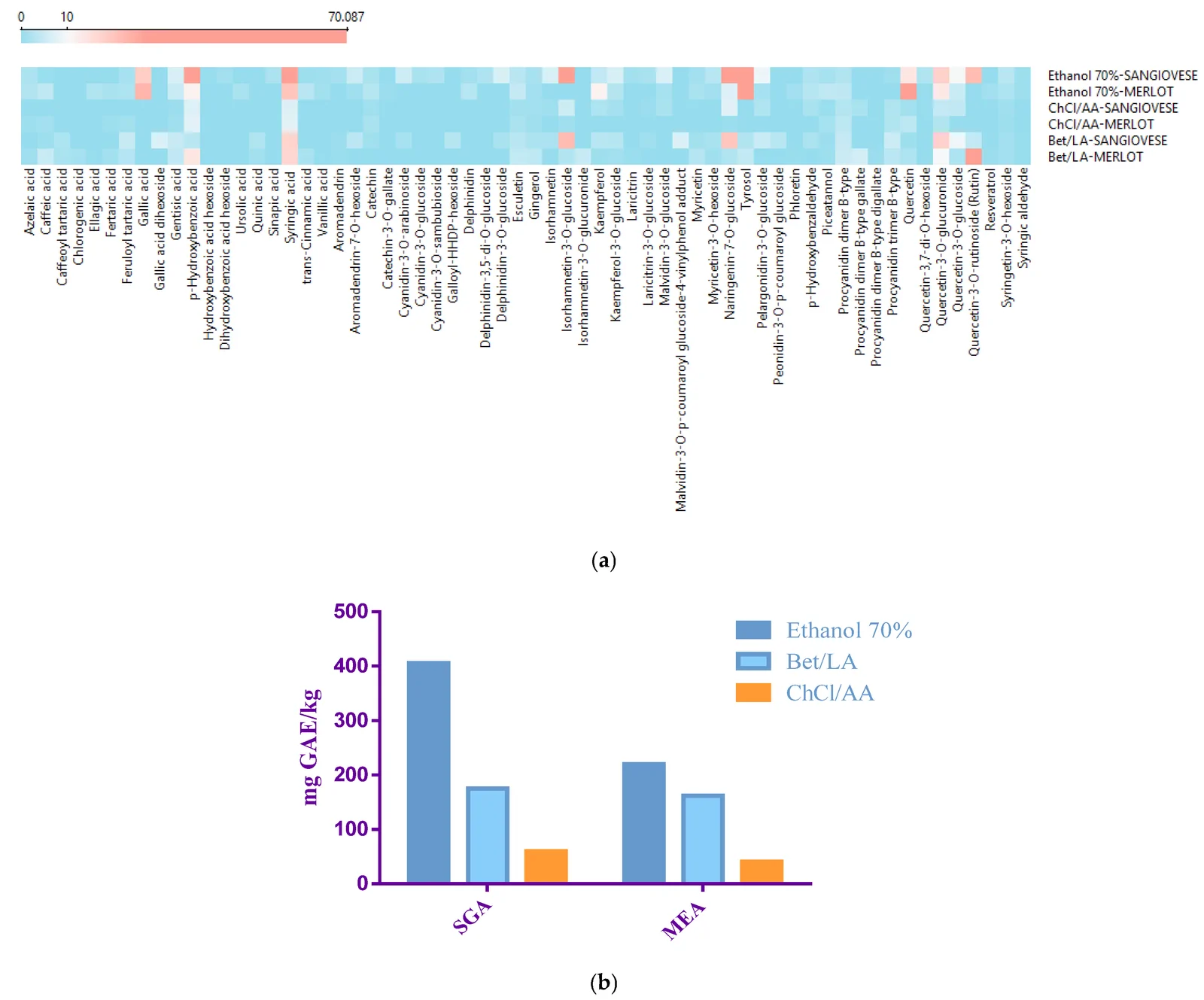
(**a**). Heatmap representation of phenolic compounds (mg GAE/kg DW) detected by LC-QTOF-MS in *Merlot* and *Sangiovese* dried grape pomace extracts (Bet/LA, ChCl/AA, and 70% ethanol). Results filtered by score up to 70%. Values are semi-quantitative, and metabolites are tentatively identified. (**b**) Total phenolic content (mg GAE/kg DW) of *Merlot* and *Sangiovese* dried grape pomace extracts (Bet/LA, ChCl/AA, and 70% ethanol), as determined by LC-QTOF-MS analysis. Values are semi-quantitative.

*Merlot* (MEA) and *Sangiovese* (SGA) dried red GPs were found to be, through HPLC-DAD analysis, among the most abundant varieties in bioactive molecules content. For this reason, their metabolomic profile was characterized in detail, with a focus on Bet/LA and ChCl/AA DESs, which were shown to be among the best extracting solvents (Table S3 and Figure 3). Other studies have investigated and demonstrated the richness and value of these varieties in terms of phytochemical content [60,61,62]. In this first analytical screening, the difference in treatment did not appear to influence or affect the phenolic content of the examined cultivars: MEA comes from an organic farming, while SGA comes from a conventional one. The dried matrices were chosen considering their potential for a higher shelf life due to the lower water content.

### 3.3. LC-QTOF-MS Qualitative and Semi-Quantitative Metabolomic Profiling

LC-QTOF-MS analysis revealed up to sixty-two metabolites, which were tentatively identified based on mass spectra and library matching (Table S3). Most of them were also detected in a recent study [28], in which HPLC-DAD-TOF was used to characterize DESs-based GP’s extracts. Semi-quantitative estimates (mg GAE/kg DW) were used to compare extraction efficiency across solvents (Table S3 and Figure 3a,b). In fact, since the authentic standards were not available for all compounds, this semi-quantitative approach was used to carry out a mere comparative metabolomic study among the solvents and the two cultivars considered. For this reason, the following results should be carefully interpreted as preliminary relative abundances rather than absolute concentrations.

In addition to Bet/LA and ChCl/AA, which resulted in the most promising DESs by HPLC-DAD analysis, ethanol 70% was kept as a reference solvent for the metabolomic characterization of *Merlot* and *Sangiovese* cultivars.

*Sangiovese* GP tended to have higher polyphenol content than *Merlot* GP across solvents. The reference ethanolic aqueous solution showed a total yield almost two-times higher for *Sangiovese* than *Merlot* GP (around 405 and 220 mg GAE/kg DW respectively). Moreover, even from a qualitative point of view, this solvent extracted more phenolic compounds (forty-six) from *Sangiovese* than from *Merlot* GP (thirty-six). Bet/LA NADES was found to be the most effective extracting green solvent: thirty-eight phenolic compounds were detected, and a total yield of 175 mg GAE/kg DW was achieved for *Sangiovese* GP, compared to thirty-six metabolites extracted and around 162 mg GAE/kg DW of yield for *Merlot* GP. ChCl/AA NADES showed the lowest results in terms of semi-quantitative extracting yields, but with twenty-three compounds and around 60 mg GAE/kg DW obtained, *Sangiovese* GP was confirmed to have a higher content of bioactive compounds than *Merlot* GP (nineteen compounds resulting in a yield of 40.4 mg GAE/kg DW). The polyphenols identified as Isorhamnetin-3-O-glucoside and Naringenin-7-O-glucoside were the most abundant metabolites found in *Sangiovese* GP in each extracting solvent, resulting in the 33.3% of the total yield obtained with ethanol 70%, the 21.2% for ChCl/AA NADES and the 23.5% for Bet/LA NADES. On the other hand, *Merlot* GP showed, varying the solvent used for the extraction, different spectra of bioactive compounds, from both a qualitative and semi-quantitative point of view: quercetin and tyrosol were found to be the most abundant metabolites in ethanol 70% (38.3 and 29.2 mg GAE/kg DW respectively), p-hydroxybenzoic and syringic acids in ChCl/AA NADES (7.6 and 7.5 mg GAE/kg DW respectively) and rutin in Bet/LA NADES (49.2 mg GAE/kg DW). Due to the tentative identification and semi-quantitative approach, the metabolomic results should be interpreted as relative trends rather than absolute quantification.

The different performances observed among extraction solvents depending on the analytical technique may be attributed to the distinct nature of the methods used. HPLC-DAD targets a limited number of specific phenolic compounds, whereas LC-QTOF-MS provides a broader overview of the metabolomic profile. Therefore, solvents such as ChCl/AA may appear more effective in targeted analysis, while Bet/LA may exhibit a greater ability to extract a wider range of metabolites. These differences highlight the importance of combining complementary analytical approaches when evaluating extraction efficiency.

### 3.4. Antimicrobial Activity

**Figure 4:**
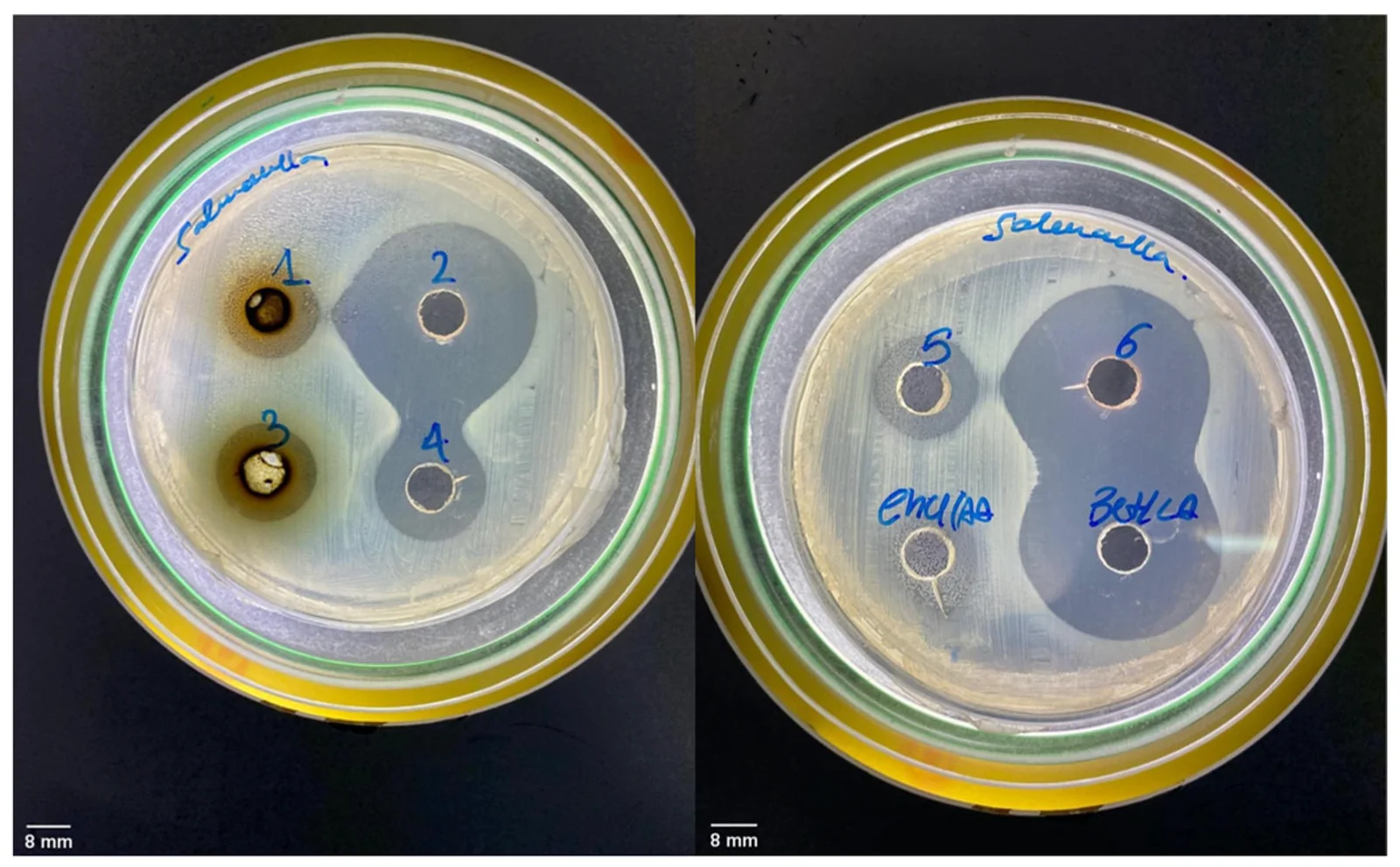
Two plates of the bacterium *Salmonella enterica* show all the extracts tested. In the plate on the left, going from the left to the right, *Merlot* ethanol 70% (1) and *Merlot* Bet/LA (2) are shown in the first line while *Sangiovese* ethanol 70% (3) and *Sangiovese* ChCl/AA (4) in the second one. In the plate on the right, going from the left to the right, *Merlot* ChCl/AA (5) and *Sangiovese* Bet/LA (6) are shown in the first line while control solvents (ChCl/AA and Bet/LA) in the second one.

**Figure 5:**
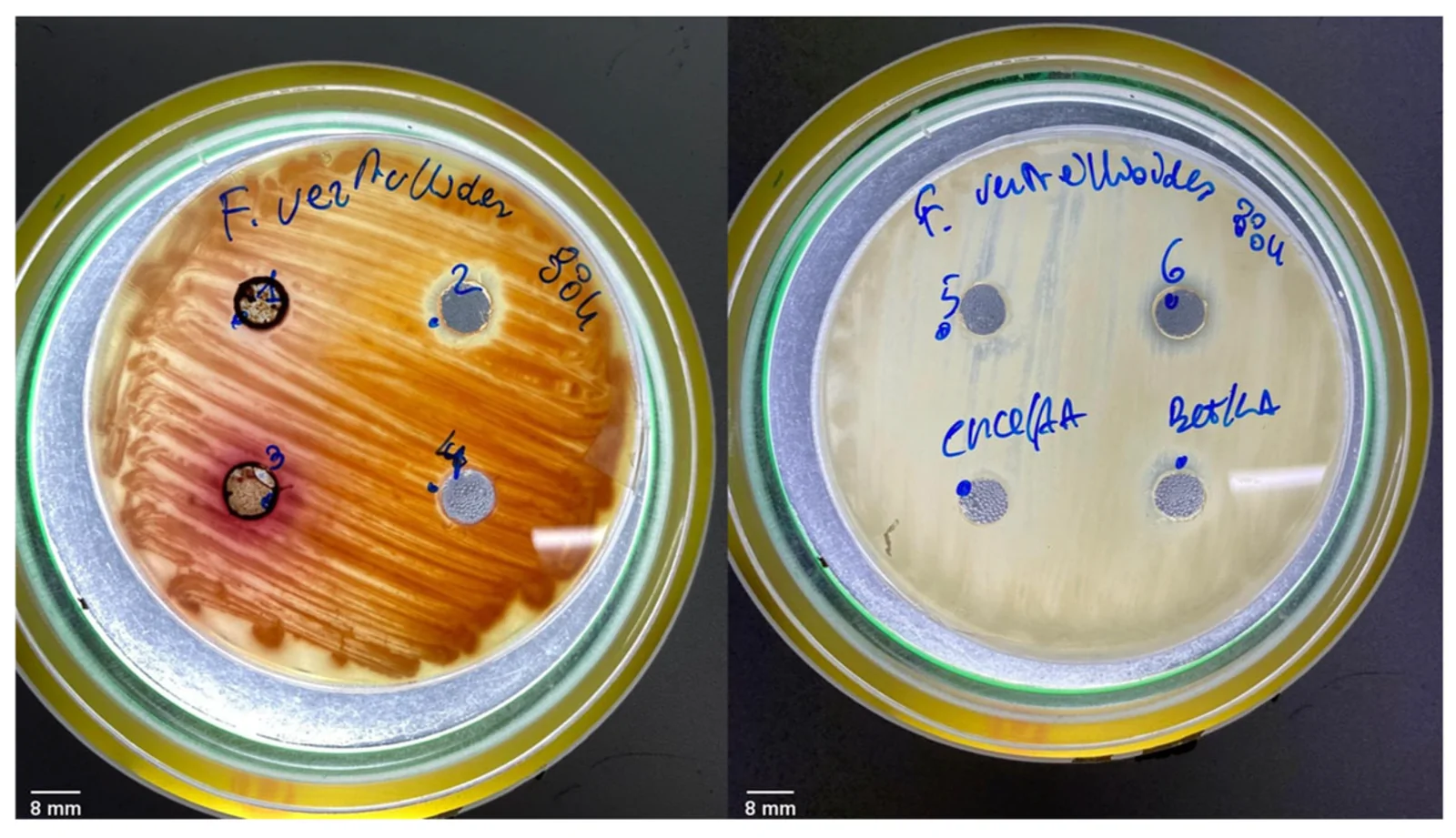
Two plates of the fungus *Fusarium verticillioides* show all the extracts tested. In the plate on the left, going from the left to the right, *Merlot* ethanol 70% (1) and *Merlot* Bet/LA (2) are shown in the first line while *Sangiovese* ethanol 70% (3) and *Sangiovese* ChCl/AA (4) in the second one. In the plate on the right, going from the left to the right, *Merlot* ChCl/AA (5) and *Sangiovese* Bet/LA (6) are shown in the first line while control solvents (ChCl/AA and Bet/LA) in the second one.

The antimicrobial activity of the same samples analytically evaluated by LC-QTOF-MS was estimated by agar diffusion assay. The results showed peculiar differences according to the microorganism and the extract considered (Table 4 and Table 5 and Figure 4 and Figure 5). This assay was based on an agar diffusion method and therefore provides only qualitative information. The observed activity may be influenced by the intrinsic properties of the NADESs, particularly their acidity, as well as by the absence of standard antibiotics or pH-adjusted controls, which represents a limitation of the study, giving it a merely qualitative meaning. The evaluation of the solvent controls under study (Bet/LA and ChCl/AA NADESs) was done as well, since previous findings have already demonstrated a certain antimicrobial activity possessed by DESs themselves against pathogenic bacteria [63,64]. Bet/LA NADES freeze-dried extracts obtained from both *Merlot* (MEA) and *Sangiovese* (SGA) GPs were the only samples, along with the control solvents, to display biological activity against pathogenic fungi: all these three samples were low effective, having an inhibition halo diameter < 8 mm, against *Fusarium verticillioides* (CECT 20926); *Botrytis allii* (CECT 2851) was also low sensible (+) to Bet/LA control solvent and *Sangiovese* Bet/LA extract while *Merlot* Bet/LA NADES extract showed an higher activity on this fungus, with an halo between 8 and 12 mm; halos < 8 mm assessed an antifungal power against *Alternaria alternata* (CECT 2662) only for Bet/LA control solvent and *Sangiovese* Bet/LA extract. Furthermore, the tested extracts were found to be active against the selected pathogenic bacteria, except *Listeria monocytogenes* (CECT 935). When effective, controls included, they showed strong activity (inhibition halos > 12 mm) against *Staphylococcus aureus* (CECT 240). *Sangiovese* and *Merlot* ethanol/water 70% extracts displayed moderate activity (++) against *Salmonella enterica* (CECT 554), and *Sangiovese*’s ethanol 70% extract showed a strong one (+++) against *E. coli* (CECT 4782). Bet/LA control solvent, as well as the corresponding extracts from both cultivars, were found to be very active (inhibition halos > 12 mm) against both *E. coli* and *S. enterica*, without any relevant differences among them. A better antibacterial efficacy on *E. coli* was evident for the ChCl/AA-based SGA extract compared to MEA and control samples’ extracts, as already assessed by the previous investigation. These results suggest antimicrobial activity associated with both the NADESs and the corresponding GP extracts, although their individual contributions cannot be clearly separated in this study.

### 3.5. Antioxidant Activity

The determination of antioxidant activity of these extracts, along with the control solvents (Table 6), added important findings to the already discussed results of the antimicrobial evaluation. Ethanol 70% control solvent was confirmed to be totally inactive, while Bet/LA NADES showed negligible residual activity (0.2 mg TEAC/g DW). Extracts from *Merlot* of both solvents displayed almost the same scavenging activity (3.2 and 3.3 mg TEAC/g DW for Bet/LA NADES and ethanol 70%, respectively), assessing the ability of the NADES to reach similar properties of the reference solvent. On the other hand, extracts from *Sangiovese* displayed slight differences with ethanolic samples, which were more active than the ones obtained with betaine-based NADES (4.3 and 2.8 mg TEAC/g DW, respectively). Ascorbic acid-based extracts, along with the control, showed the highest, but all comparable, values: in this case, the contribution of the polyphenols extracted to the total resulting bioactivity was negligible. No improvements over the control were noticed, since ascorbic acid itself, as the starting ingredient of this NADES, strongly influenced the results of the assay. This result showed that the antioxidant activity of ChCl/AA-based samples is mostly due to the formulation of the NADES rather than the polyphenols extracted, which, on the other hand, clearly had a higher impact in Bet/LA-based extracts. This confirmed the findings obtained by LC-QTOF-MS, in which the extraction ability of Bet/LA over ChCl/AA-based extracts was assessed, but underlined the potentialities that ascorbic acid-based formulations display in terms of scavenging impact.

### 3.6. Total Polyphenol Content

To give more insides about antioxidant activity potential, the Folin–Ciocalteu assay was performed, although it was expected that AA would be responsible for the bulk of the observed antioxidant activity. The quantification of total polyphenols (Table 7) showed minor differences between the two cultivars assessing an almost equal content of phenolics for each extracting solvent employed. As observed with the detection of antioxidant activity, ethanol 70% and Bet/LA NADES control solvents were again confirmed to have no contribution to the resulting polyphenol dosage. Bet/LA-based extracts displayed a higher total polyphenol content (around 0.90 mg GAE/g DW) than ethanolic extracts (around 0.60 mg GAE/g DW).

Ascorbic acid-based NADES control solvent showed a total content of polyphenols around 3.31 mg GAE/g DW: a value that makes evident that the contribution of this extracting media cannot be ignored. Its extracts from both cultivars showed comparable results (3.38 and 3.20 mg GAE/g DW) for *Sangiovese* and *Merlot*, respectively) since ascorbic acid, as a major ingredient of the samples, contributed notably to the measured polyphenol content. Moreover, this evidence confirmed, as already seen with the antimicrobial activity shown by Bet/LA NADES control solvent, the significant contribution of these new green solvents. Although their effects make it difficult to give an unequivocal attribution of the role played by the numerous polyphenols recovered from by-products and identified through the LC-QTOF-MS technique, they show further meaningful properties that make them substantial and edible ingredients as well in possible future applications.

## 4. Conclusions

Three different NADESs were used as alternative green liquid extracting phases to recover bioactive compounds from seventeen different varieties of grape pomaces collected in Apulia (Italy). An initial screening of their content in polyphenols was performed by HPLC-DAD. This qualitative–quantitative analysis showed that organic *Merlot* (MEA) and conventional *Sangiovese* (SGA) extracted with Bet/LA and ChCl/AA NADESs were the most interesting and promising samples. A complete semi-quantitative metabolomic characterization of these selected extracts was carried out through LC-QTOF-MS: betaine-based NADES, aside from ethanol/water 70% used as reference, was found to be the most effective solvent for both cultivars compared to ChCl/AA NADES. Qualitative antimicrobial assays, performed on the same samples through agar diffusion technique, showed a significant activity of Bet/LA (MEA) and ChCl/AA (SGA) extracts on *B. allii* and *E. coli*, respectively, although it is difficult to discriminate the contribution of NADESs and GPs polyphenols to the observed effects. In the latter case, this is a confirmation of the trend found with the ethanolic extracts. In several cases, there was no relevant difference in the activity within extracts and NADES themselves. The detection of the scavenging activity and total polyphenols confirmed, even in this case, better results of NADES extracts and control solvent over ethanolic ones.

Moreover, although the content of ascorbic acid, used as an ingredient of the solvent, substantially influenced the relevant obtained results in ChCl/AA samples, the observed results for Bet/LA extracts in both assays indicate the presence of antioxidant activity and suggest potential for further optimization and application-oriented investigation. However, the observation that, in some cases, control solvents exhibited equal or even higher antimicrobial and/or antioxidant activities than the extracts should be interpreted within the intended application of NADESs. In formulations designed for such mentioned ready-to-use applications, NADESs are not merely extraction media but integral components of the final product, contributing to bioactivity while simultaneously enabling the extraction and delivery of polyphenols. Further studies will be carried out to deepen the evolution of the metabolomic profile as well as the biological activity during a simulated digestion process, with the aim of assessing a possible future application of these extracts for the formulation of feed and/or food supplements.

## Figures and Tables

**Table 1:** Grape pomace varieties under study, classified according to treatment type, origin and abbreviation.

Grape Variety	Treatment Type	Origin	Abbreviation
*Merlot*	Organic	Andria (Barletta-Andria-Trani)	MEA
*Syrah*	First year of organic conversion	Andria (Barletta-Andria-Trani)	SYA
*Minutolo*	Organic	Ruvo di Puglia (Bari)	MIR
*Trebbiano*	Conventional	Andria (Barletta-Andria-Trani)	TRA
*Primitivo*	Conventional	Ruvo di Puglia (Bari)	PRA
*Moscato*	Conventional	Andria (Barletta-Andria-Trani)	MOA
*Sangiovese*	Conventional	Andria (Barletta-Andria-Trani)	SGA
*Nero di Troia*	Organic	Ruvo di Puglia (Bari)	NTR
*Negro Amaro*	Conventional	Manduria (Taranto)	NAT
*Nero di Troia*	Conventional	Corato (Bari)	NTC
*Nero di Troia*	Organic	Andria (Barletta-Andria-Trani)	NTA
*Montepulciano*	Conventional	Minervino Murge (Barletta-Andria-Trani)	MOM
*Primitivo*	Conventional	Turi (Bari)	PRB
*Primitivo*	Conventional	Manduria (Taranto)	PRT
*Montepulciano*	Organic	Andria (Barletta-Andria-Trani)	MTA
*Malbec*	Biodynamic	Manduria (Taranto)	MAM
*Negro Amaro*	Biodynamic	Manduria (Taranto)	NAM

**Table 2:** Natural deep eutectic solvents’ composition.

HBA	HBD	Molar Ratio	Water Content (%)	Reference
ChCl	AA	2:1	40	[35]
Bet	LA	1:4	40	[36]
ChCl	Urea	1:2	40	[37]

HBA, hydrogen-bonding acceptor; HBD, hydrogen-bonding donor.

**Table 3:** Chemical composition of the grape pomace varieties under study, expressed as percentage (%) of dry matter (mean ± standard error; all determinations were conducted in triplicate).

Grape Pomace	Moisture	Crude Protein	Crude Fiber	Crude Lipids	Total Ash
MEA	35.36 ± 3.22	11.88 ± 0.69	42.57 ± 1.76	6.42 ± 0.88	6.71 ± 0.21
SYA	48.25 ± 2.71	11.19 ± 0.59	27.35 ± 1.11	2.87 ± 0.39	9.14 ± 0.25
MIR	57.46 ± 3.01	11.19 ± 0.61	25.44 ± 1.54	7.93 ± 0.80	5.56 ± 0.19
TRA	55.64 ± 2.97	13.94 ± 0.63	33.92 ± 1.60	2.32 ± 0.32	5.30 ± 020
PRA	50.09 ± 3.13	13.75 ± 0.70	33.20 ± 1.49	6.74 ± 0.79	7.02 ± 0.18
MOA	46.78 ± 3.88	7.22 ± 0.55	12.53 ± 0.98	3.00 ± 0.62	7.17 ± 0.18
SGA	59.44 ± 4.02	12.69 ± 0.51	27.72 ± 1.11	4.00 ± 0.66	9.18 ± 0.20
NTR	64.56 ± 4.15	11.06 ± 0.53	24.48 ± 1.02	3.63 ± 0.58	6.46 ± 0.15
NAT	68.46 ± 4.07	11.34 ± 0.42	29.61 ± 1.13	2.98 ± 0.40	7.60 ± 0.19
NTC	40.74 ± 3.47	10.69 ± 0.40	28.30 ± 1.23	4.61 ± 0.33	6.06 ± 0.17
NTA	51.74 ± 3.66	10.94 ± 0.39	32.87 ± 1.37	3.45 ± 0.29	7.77 ± 0.21
MOM	55.31 ± 3.95	11.22 ± 0.41	32.17 ± 1.40	3.30 ± 0.31	6.03 ± 0.19
PRB	58.20 ± 3.63	10.09 ± 0.40	24.47 ± 1.12	3.33 ± 0.35	8.11 ± 0.24
PRT	60.56 ± 4.14	10.97 ± 0.39	21.84 ± 1.03	3.44 ± 0.29	10.35 ± 0.27
MTA	51.05 ± 3.86	11.94 ± 0.38	24.97 ± 1.07	4.00 ± 0.38	10.32 ± 0.26
MAM	48.33 ± 3.05	11.84 ± 0.38	23.90 ± 1.10	3.53 ± 0.41	11.44 ± 0.29
NAM	66.60 ± 4.48	10.19 ± 0.29	25.67 ± 1.24	3.42 ± 0.44	10.41 ± 0.27

**Table 4:** Qualitative determination of antifungal activity according to agar diffusion assay.

Fungi	Strain	EtOH 70–MEA	EtOH 70%-SGA	Bet/LA	Bet/LA-MEA	Bet/LA-SGA	ChCl/AA	ChCl/AA-MEA	ChCl/AA-SGA
*F. verticillioides*	CECT 20926	−	−	+	+	+	−	−	−
*A. flavus*	ISPA 8111	−	−	−	−	−	−	−	−
*B. cinerea*	CECT 2100	−	−	−	−	−	−	−	−
*B. allii*	CECT 2851	−	−	+	++	+	−	−	−
*A. alternata*	CECT 2662	−	−	+	−	+	−	−	−

(−) no inhibition halo; (+) mean diameter inhibition halo < 8 mm; (++) mean diameter inhibition halo 8–12 mm; (+++) mean diameter inhibition halo > 12 mm.

**Table 5:** Qualitative determination of antibacterial activity according to agar diffusion assay.

Bacteria	Strain	EtOH 70%-MEA	EtOH 70%-SGA	Bet/LA	Bet/LA-MEA	Bet/LA-SGA	ChCl/AA	ChCl/AA-MEA	ChCl/AA-SGA
*S. aureus*	CECT 240	+++	+++	+++	+++	+++	+++	+++	+++
*E. coli*	CECT 4782	++	+++	+++	+++	+++	++	++	+++
*L. monocytogenes*	CECT 935	−	−	−	−	−	−	−	−
*S. enterica*	CECT 554	++	++	+++	+++	+++	++	++	++

(−) no inhibition halo; (+) mean diameter inhibition halo < 8 mm; (++) mean diameter inhibition halo 8–12 mm; (+++) mean diameter inhibition halo > 12 mm.

**Table 6:** Detection of antioxidant activity according to ABTS radical cation decolorization assay.

Sample	Antioxidant Activity (mg TEAC/g DW) ^a^
Bet/LA	0.2 ± 0.2
Bet/LA-SGA	2.8 ± 0.1
Bet/LA-MEA	3.2 ± 0.1
EtOH 70%	ND
EtOH 70-SGA	4.3 ± 0.1
EtOH 70-MEA	3.3 ± 0.1
ChCl/AA	2074.9 ± 234.7
ChCl/AA-SGA	1915.2 ± 290.4
ChCl/AA-MEA	1770.0 ± 0.1

ND, not detectable; (^a^) values as mean ± SD (*n* = 2).

**Table 7:** Detection of total polyphenols according to the Folin–Ciocalteu assay.

Sample	Total Polyphenols (mg GAE/g DW) ^a^
Bet/LA	ND
Bet/LA-SGA	0.92 ± 0.02
Bet/LA-MEA	0.93 ± 0.02
EtOH 70%	ND
EtOH 70–SGA	0.58 ± 0.03
EtOH 70–MEA	0.55 ± 0.02
ChCl/AA	3.31 ± 0.05
ChCl/AA-SGA	3.38 ± 0.16
ChCl/AA-MEA	3.20 ± 0.02

ND, not detectable; (^a^) values as mean ± SD (*n* = 3).

## Data Availability

The original contributions presented in this study are included in the article/Supplementary Material. Further inquiries can be directed to the corresponding author.

## Supplementary Materials

The following supporting information can be downloaded at: https://www.mdpi.com/article/10.3390/molecules31101665/s1, Table S1: Phenolic compounds (mg/kg DW) detected in all extracts from fresh GPs according to HPLC-DAD analysis. Figure S1: Content of catechin (mg/kg DW) detected in all extracts from fresh GPs according to HPLC-DAD analysis. Figure S2: Content of rutin (mg/kg DW) detected in all extracts from fresh GPs according to HPLC-DAD analysis. Figure S3: Content of quercetin (mg/kg DW) detected in all extracts from fresh GPs according to HPLC-DAD analysis. Table S2: Phenolic compounds (mg/kg DW) detected in all extracts from dried GPs according to HPLC-DAD analysis. Figure S4: Content of catechin (mg/kg DW) detected in all extracts from dried GPs according to HPLC-DAD analysis. Figure S5: Content of rutin (mg/kg DW) detected in all extracts from dried GPs according to HPLC-DAD analysis. Figure S6: Content of quercetin (mg/kg DW) detected in all extracts from dried GPs according to HPLC-DAD analysis. Table S3: Phenolic compounds (mg GAE/kg DW) detected in *Sangiovese* (SGA) and *Merlot* (MEA) GPs extracted with ethanol 70% (wt/wt), Bet/LA and ChCl/AA NADESs, according to LC-QTOF-MS analysis.
