# Supplementary material for: Edible Natural Deep Eutectic Solvents (NADESs)-Based Polyphenolic Extracts: An Eco-Sustainable Alternative for Grape Pomace Valorization
Source: Molecules. 2026 May 15;31(10):1665. doi: 10.3390/molecules31101665 (PMC13209698; doi:10.3390/molecules31101665)
Supplement: Supplementary file 1 [file molecules-31-01665-s001.zip › molecules-4226269-supplementary.pdf]

Table S1. Phenolic compounds (mg/kg DW) detected in all extracts from fresh GPs according to HPLC-DAD analysis. ([a])Values as mean ± SD (n = 2).

GRAPE POMACE	SOLVENT	Gallic acid (mg/kg) [a]	Syringic acid (mg/kg) [a]	Coumaric acid (mg/kg) [a]	Kaempferol-3-O-galactoside (mg/kg) [a]	Chlorogenic acid (mg/kg) [a]	Resveratrol (mg/kg) [a]	Ferulic acid (mg/kg) [a]	Caffeic acid (mg/kg) [a]	Catechin (mg/kg) [a]	Rutin (mg/kg) [a]	Quercetin (mg/kg) [a]
MEA	Ethanol/water 70%	13.8±0.7	5.5±0.8	0.4±0.5	ND	6.1±7.7	0.4±0.0	0.1±0.2	ND	46.4±10.7	108.0±8.9	329.1±2.1
	ChCl/urea	26.8±0.2	3.7±0.4	0.3±0.0	ND	ND	ND	0.2±0.2	ND	53.0±3.7	61.2±2.6	135.2±3.8
	Bet/LA	22.9±3.4	4.5±1.0	0.3±0.4	ND	ND	ND	0.4±0.0	ND	41.9±15.0	31.1±5.0	115.2±8.3
	ChCl/AA	16.0±1.6	2.2±0.3	0.3±0.0	ND	ND	ND	0.3±0.0	ND	45.2±5.5	51.6±9.8	163.6±6.0
SYA	Ethanol/water 70%	ND	6.4±2.4	ND	ND	ND	ND	ND	ND	ND	94.2±6.0	ND
	ChCl/urea	10.8±0.9	4.8±0.7	ND	ND	ND	ND	ND	ND	<LOQ	43.0±9.1	ND
	Bet/LA	8.5±0.3	6.9±0.6	ND	ND	ND	ND	ND	ND	22.8±0.2	ND	ND
	ChCl/AA	6.8±0.0	3.0±0.2	ND	ND	ND	ND	ND	ND	46.6±7.4	ND	ND
MIR	Ethanol/water 70%	7.8±2.0	ND	ND	ND	ND	ND	ND	ND	ND	10.6±3.1	ND
	ChCl/urea	ND	ND	ND	ND	ND	ND	ND	ND	<LOQ	19.1±9.1	ND
	Bet/LA	6.9±1.3	ND	ND	ND	ND	ND	ND	ND	27.4±0.3	9.2±0.5	ND
	ChCl/AA	6.5±0.1	ND	ND	ND	ND	ND	ND	ND	246.9±4.3	9.2±2.5	ND
TRA	Ethanol/water 70%	ND	ND	ND	ND	ND	ND	0.4±0.0	ND	ND	48.3±15.7	ND
	ChCl/urea	ND	ND	ND	ND	ND	ND	0.3±0.0	ND	ND	44.9±8.0	ND
	Bet/LA	4.7±0.2	ND	ND	ND	ND	ND	0.4±0.0	ND	26.5±4.7	61.9±2.9	ND
	ChCl/AA	5.2±0.3	ND	ND	ND	ND	ND	ND	ND	243.2±36.4	55.3±24.0	ND
PRA	Ethanol/water 70%	ND	6.2±1.0	ND	ND	15.0±0.9	ND	ND	ND	32.6±8.6	39.6±0.3	ND
	ChCl/urea	15.4±1.8	13.6±2.4	ND	ND	ND	0.5±0.2	ND	0.4±0.0	37.5±11.1	ND	ND
	Bet/LA	14.7±0.2	5.4±1.0	ND	ND	ND	0.4±0.0	ND	ND	56.0±8.6	33.0±3.8	ND
	ChCl/AA	9.7±0.1	5.8±1.0	ND	ND	ND	ND	ND	ND	232.9±31.1	37.5±7.9	ND
MOA	Ethanol/water 70%	ND	ND	ND	<LOQ	ND	ND	ND	ND	ND	17.2±5.3	ND
	ChCl/urea	6.5±0.3	ND	ND	ND	ND	ND	ND	0.4±0.0	ND	16.1±3.7	ND
	Bet/LA	4.7±0.2	ND	ND	<LOQ	ND	ND	0.2±0.2	0.2±0.2	23.7±0.9	19.7±5.9	ND
	ChCl/AA	ND	ND	ND	<LOQ	ND	0.4±0.5	0.3±0.0	ND	412.7±9.6	19.2±0.4	ND
SGA	Ethanol/water 70%	3.2±4.4	2.3±0.0	ND	ND	ND	ND	ND	ND	<LOQ	163.5±13.5	57.4±73.9
	ChCl/urea	17.8±4.9	3.1±1.5	0.2±0.3	<LOQ	ND	ND	ND	ND	<LOQ	165.9±66.8	ND
	Bet/LA	6.8±1.7	<LOQ	ND	<LOQ	ND	ND	ND	ND	37.4±11.8	191.1±177.5	ND
	ChCl/AA	5.9±0.3	<LOQ	ND	ND	ND	ND	0.3±0.1	ND	478.9±60.1	185.9±40.6	61.0±79.0
NTR	Ethanol/water 70%	ND	2.5±0.2	ND	ND	ND	0.6±0.1	ND	ND	ND	170.7±31.4	ND
	ChCl/urea	24.2±11.1	3.7±2.2	ND	ND	5.9±7.4	ND	ND	ND	<LOQ	65.1±6.9	ND
	Bet/LA	4.7±0.1	<LOQ	ND	ND	ND	ND	ND	ND	41.0±1.2	157.7±0.9	ND
	ChCl/AA	ND	ND	0.6±0.0	ND	ND	ND	ND	ND	553.2±13.1	108.5±15.2	ND

NAT	Ethanol/water 70%	5.3±0.6	<LOQ	ND	ND	ND	ND	ND	ND	ND	8.9±3.7	ND
	ChCl/urea	12.0±0.4	2.4±0.1	ND	ND	ND	ND	ND	ND	<LOQ	18.0±2.4	ND
	Bet/LA	9.6±0.4	<LOQ	ND	ND	ND	ND	ND	ND	32.9±0.9	13.9±2.9	ND
	ChCl/AA	9.6±0.4	<LOQ	ND	ND	ND	ND	ND	ND	341.2±13.6	13.2±1.2	ND
NTC	Ethanol/water 70%	ND	6.1±0.2	ND	ND	ND	0.4±0.0	ND	ND	21.3±0.9	149.4±24.8	211.1±17.0
	ChCl/urea	44.0±2.5	8.2±0.7	ND	ND	ND	0.4±0.0	ND	ND	30.3±0.5	38.0±2.4	ND
	Bet/LA	13.4±0.0	4.9±0.0	ND	ND	ND	ND	ND	ND	52.1±1.8	147.0±2.2	ND
	ChCl/AA	10.6±0.8	3.5±0.2	0.5±0.0	ND	ND	ND	ND	ND	367.6±9.7	115.7±1.3	198.9±16.8
NTA	Ethanol/water 70%	10.3±1.5	3.4±0.2	ND	ND	ND	ND	ND	ND	22.0±4.6	ND	136.5±7.7
	ChCl/urea	20.2±0.8	4.5±0.3	ND	ND	ND	ND	ND	ND	27.0±1.8	ND	ND
	Bet/LA	15.1±0.6	3.4±0.3	0.9±0.1	ND	ND	ND	ND	ND	36.7±0.1	ND	ND
	ChCl/AA	11.8±0.4	<LOQ	0.3±0.4	ND	ND	0.9±0.1	0.6±0.1	ND	106.9±14.3	ND	232.6±16.0
MOM	Ethanol/water 70%	8.9±0.3	2.8±0.7	0.9±0.2	ND	ND	0.9±0.4	ND	ND	19.9±0.3	ND	460.4±66.5
	ChCl/urea	26.3±2.2	3.6±1.8	ND	ND	ND	0.2±0.2	ND	ND	24.7±1.1	ND	61.6±79.8
	Bet/LA	17.8±0.8	2.7±0.7	1.3±0.5	ND	ND	0.2±0.2	ND	ND	42.8±3.0	ND	80.2±106.2
	ChCl/AA	10.0±1.0	2.0±0.2	ND	10.8±1.0	ND	0.8±0.1	ND	ND	159.2±12.1	ND	112.2±9.6
PRB	Ethanol/water 70%	ND	6.8±0.4	ND	ND	ND	ND	0.3±0.0	ND	<LOQ	ND	ND
	ChCl/urea	9.1±0.1	7.7±0.8	ND	ND	ND	ND	0.3±0.0	ND	<LOQ	ND	ND
	Bet/LA	8.3±0.3	11.2±0.4	0.7±0.0	ND	ND	ND	0.4±0.0	ND	29.8±1.9	ND	ND
	ChCl/AA	6.8±0.3	5.7±0.8	ND	ND	ND	0.7±0.0	0.4±0.0	ND	143.6±5.7	ND	ND
PRT	Ethanol/water 70%	5.9±0.2	6.0±0.7	ND	ND	ND	ND	ND	ND	<LOQ	ND	ND
	ChCl/urea	9.8±0.2	5.8±0.2	ND	ND	ND	ND	ND	ND	<LOQ	ND	ND
	Bet/LA	9.2±0.8	7.1±0.7	ND	ND	ND	ND	ND	ND	27.9±1.3	ND	ND
	ChCl/AA	7.1±0.4	5.2±0.4	ND	ND	ND	0.2±0.3	ND	ND	155.0±6.7	ND	ND
MTA	Ethanol/water 70%	12.8±0.9	3.2±0.4	ND	49.8±0.2	ND	ND	ND	ND	23.9±1.2	8.6±0.6	342.9±40.9
	ChCl/urea	35.4±0.2	3.8±0.1	ND	42.2±1.7	ND	ND	ND	ND	24.7±0.2	8.6±0.7	124.7±9.1
	Bet/LA	23.3±0.2	3.2±0.1	1.2±0.0	43.8±1.8	ND	ND	ND	ND	31.8±0.3	10.9±0.7	ND
	ChCl/AA	19.1±1.6	2.6±0.1	ND	41.6±5.5	18.4±0.8	1.5±0.5	ND	ND	41.3±10.9	ND	224.7±12.6
MAM	Ethanol/water 70%	ND	5.3±0.4	ND	ND	ND	ND	ND	ND	ND	6.6±0.6	ND
	ChCl/urea	8.1±1.3	4.1±2.1	ND	ND	ND	ND	ND	ND	<LOQ	<LOQ	ND
	Bet/LA	8.5±0.6	4.1±0.1	0.8±0.1	ND	ND	ND	ND	ND	30.9±1.2	8.6±0.4	ND
	ChCl/AA	6.4±0.2	2.2±0.1	ND	ND	11.7±0.0	1.1±0.0	ND	ND	32.9±3.7	ND	ND
NAM	Ethanol/water 70%	ND	<LOQ	ND	ND	ND	ND	ND	ND	ND	ND	ND
	ChCl/urea	5.6±0.0	ND	ND	ND	ND	ND	ND	ND	<LOQ	ND	ND
	Bet/LA	6.2±0.2	ND	ND	ND	ND	ND	ND	ND	22.7±1.0	ND	ND
	ChCl/AA	5.4±0.0	ND	ND	ND	ND	ND	ND	ND	58.6±4.9	ND	ND

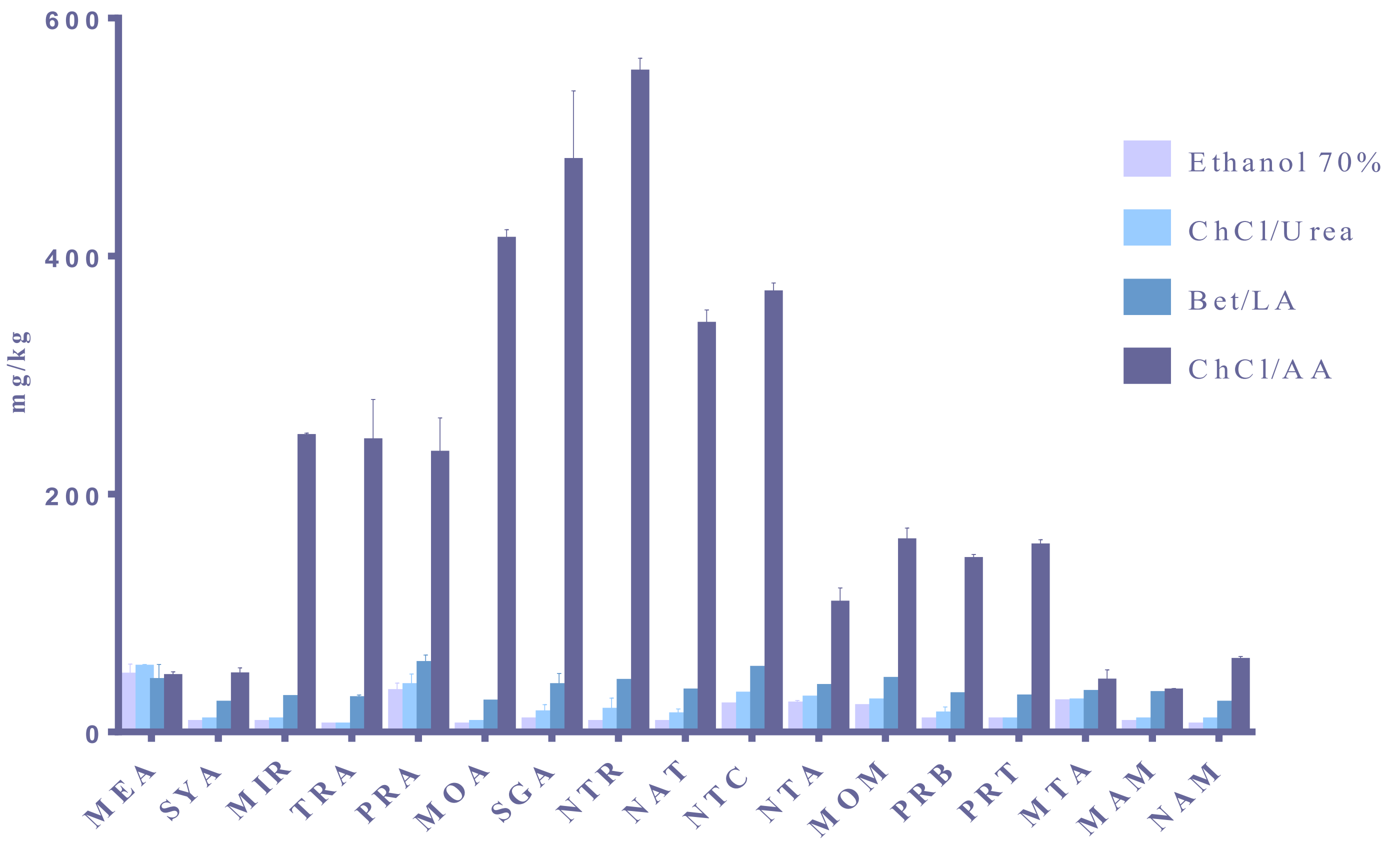

Figure S1. Content of catechin (mg/kg DW) detected in all extracts from fresh GPs according to HPLC-DAD analysis.

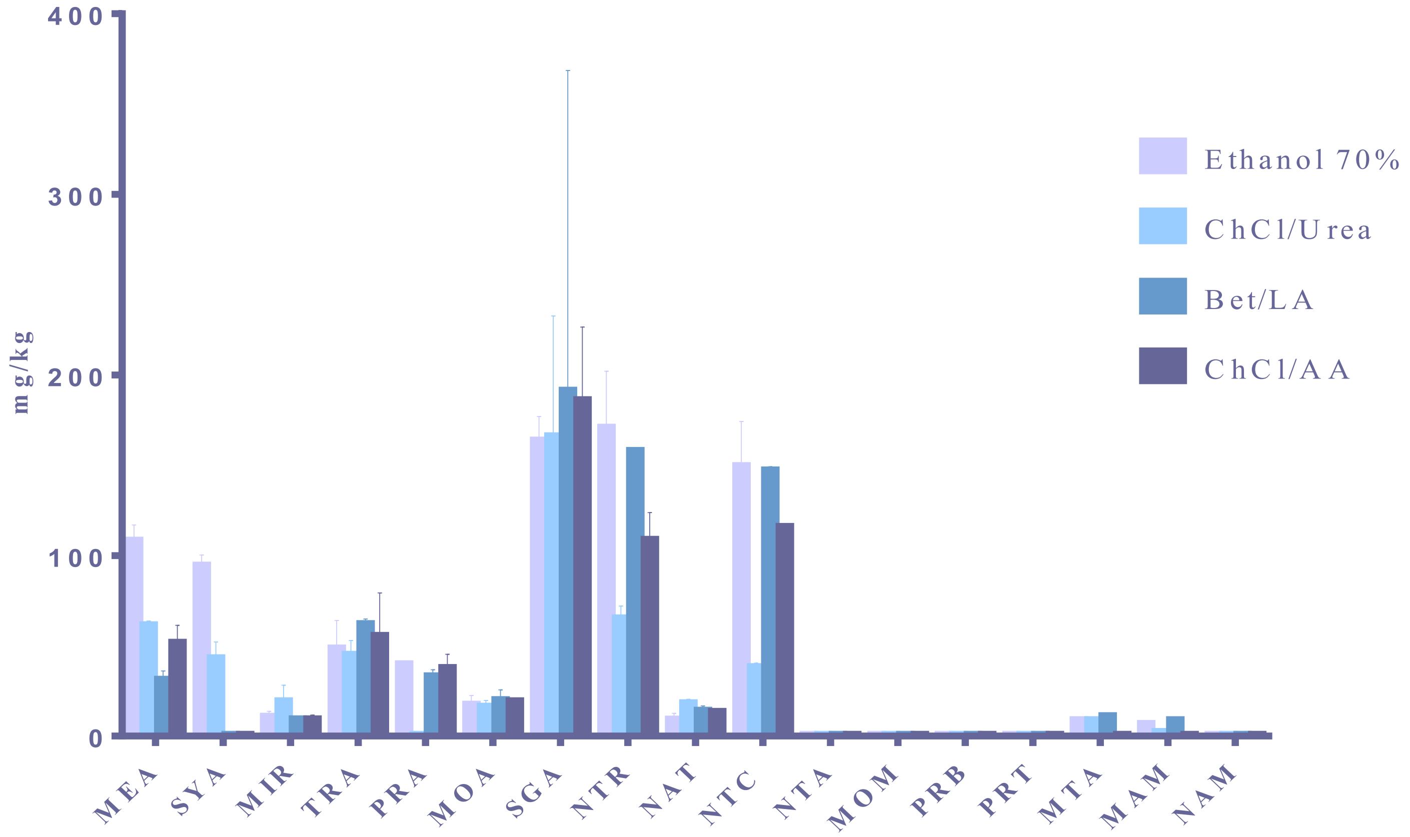

Figure S2. Content of rutin (mg/kg DW) detected in all extracts from fresh GPs according to HPLC-DAD analysis.

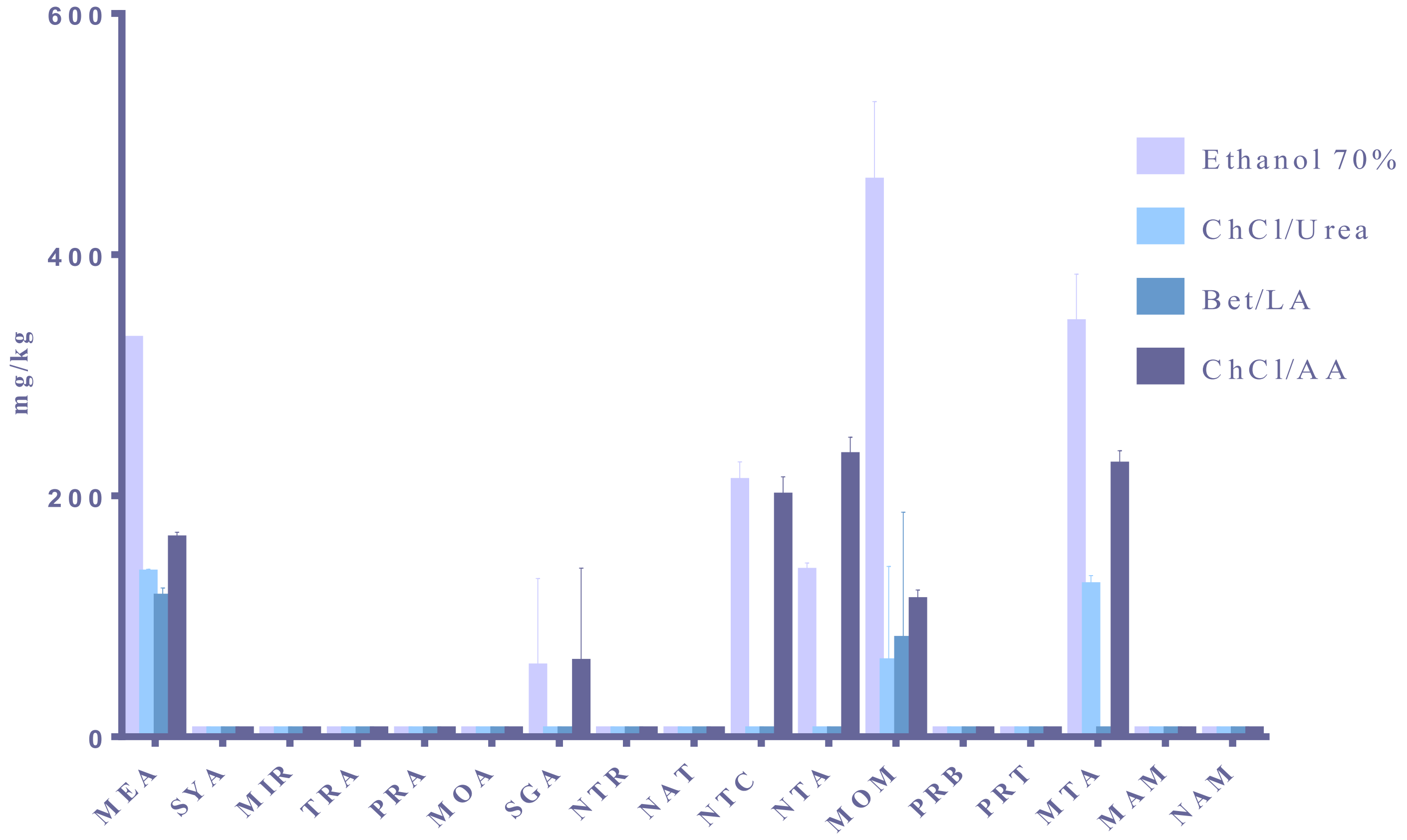

Figure S3. Content of quercetin (mg/kg DW) detected in all extracts from fresh GPs according to HPLC-DAD analysis.

Table S2. Phenolic compounds (mg/kg DW) detected in all extracts from dried GPs according to HPLC-DAD analysis. ([a])Values as mean ± SD (n = 2).

GRAPE POMACE	SOLVENT	Gallic acid (mg/kg) [a]	Syringic acid (mg/kg) [a]	Coumaric acid (mg/kg) [a]	Kaempferol-3-O-galactoside (mg/kg) [a]	Chlorogenic acid (mg/kg) [a]	Resveratrol (mg/kg) [a]	Ferulic acid (mg/kg) [a]	Caffeic acid (mg/kg) [a]	Catechin (mg/kg) [a]	Rutin (mg/kg) [a]	Quercetin (mg/kg) [a]
MEA	Ethanol/water 70%	23.2±1.0	8.6±0.2	0.5±0.1	ND	11.7±0.1	0.4±0.1	0.4±0.0	ND	68.7±0.1	115.8±8.1	405.9±33.5
	ChCl/urea	48.2±2.4	7.7±0.1	0.7±0.2	ND	ND	0.3±0.0	0.2±0.2	ND	94.7±2.3	57.7±11.4	148.0±3.9
	Bet/LA	46.5±2.3	13.5±0.7	0.6±0.1	ND	ND	0.4±0.0	0.3±0.3	ND	101.0±8.9	29.9±4.2	150.4±4.1
	ChCl/AA	24.1±0.5	5.6±0.4	0.5±0.1	ND	ND	ND	0.4±0.0	ND	457.5±9.2	69.6±0.3	201.1±8.9
SYA	Ethanol/water 70%	6.8±0.2	15.4±0.5	ND	ND	ND	ND	ND	ND	17.5±0.1	ND	129.8±5.1
	ChCl/urea	7.3±0.5	6.9±0.8	ND	ND	ND	ND	ND	ND	<LOQ	ND	ND
	Bet/LA	7.0±0.2	10.3±02	ND	ND	ND	ND	ND	ND	<LOQ	ND	ND
	ChCl/AA	ND	4.2±1.0	ND	ND	ND	ND	ND	ND	187.3±62.7	ND	ND
MIR	Ethanol/water 70%	13.4±0.6	ND	ND	ND	ND	ND	0.2±0.2	ND	<LOQ	12.7±4.8	ND
	ChCl/urea	14.2±0.5	ND	ND	ND	ND	ND	0.2±0.2	ND	<LOQ	8.5±0.7	ND
	Bet/LA	20.4±2.9	ND	ND	ND	ND	ND	0.2±0.2	ND	24.9±0.9	21.0±1.2	ND
	ChCl/AA	11.2±0.2	ND	ND	ND	ND	ND	ND	ND	575.9±9.0	14.9±3.2	ND
TRA	Ethanol/water 70%	7.6±1.7	ND	ND	ND	ND	ND	0.4±0.0	ND	ND	ND	124.4±10.6
	ChCl/urea	7.4±0.4	ND	ND	ND	ND	ND	ND	ND	<LOQ	4.3±0.8	ND
	Bet/LA	8.0±0.1	ND	ND	ND	ND	ND	0.2±0.2	ND	<LOQ	ND	ND
	ChCl/AA	7.6±0.2	ND	ND	ND	ND	ND	ND	ND	128.2±5.0	44.2±24.2	65.3±85.1
PRA	Ethanol/water 70%	12.9±0.2	35.5±6.8	ND	ND	12.0±0.1	ND	ND	ND	37.7±6.5	ND	ND
	ChCl/urea	21.5±0.9	28.4±0.9	ND	ND	ND	ND	ND	ND	46.7±1.0	ND	ND
	Bet/LA	19.0±1.9	27.2±2.3	0.9±0.1	ND	ND	0.2±0.2	ND	ND	47.0±2.5	ND	ND
	ChCl/AA	17.2±1.5	29.4±0.8	ND	ND	ND	1.0±0.4	ND	ND	321.0±195.6	ND	ND
MOA	Ethanol/water 70%	13.0±1.1	ND	ND	ND	ND	ND	0.6±0.0	0.5±0.0	ND	34.1±7.3	128.8±2.1
	ChCl/urea	10.2±0.7	ND	ND	ND	ND	ND	0.4±0.1	ND	ND	6.0±0.5	ND
	Bet/LA	11.5±2.0	ND	ND	ND	ND	ND	0.2±0.3	ND	<LOQ	ND	ND
	ChCl/AA	7.9±0.9	ND	ND	ND	ND	ND	ND	ND	90.5±5.1	11.0±5.1	ND
SGA	Ethanol/water 70%	13.4±1.0	12.3±1.1	ND	ND	ND	ND	ND	ND	42.2±1.7	289.0±30.9	161.6±2.8
	ChCl/urea	21.4±3.8	7.3±1.0	ND	ND	ND	ND	ND	ND	51.0±3.8	206.8±43.2	ND
	Bet/LA	16.5±0.6	7.0±1.1	ND	ND	ND	ND	ND	ND	64.3±3.5	249.1±54.9	ND
	ChCl/AA	14.3±2.0	4.8±0.2	ND	ND	ND	ND	ND	ND	138.8±0.2	255.6±57.7	138.5±14.7
NTR	Ethanol/water 70%	25.6±4.2	27.5±2.2	ND	ND	ND	0.4±0.0	ND	ND	54.7±3.5	174.2±27.4	339.2±12.8
	ChCl/urea	36.7±4.2	19.3±1.6	ND	ND	ND	ND	ND	0.4±0.1	61.0±2.0	47.5±13.4	ND
	Bet/LA	27.2±0.2	17.7±1.8	ND	ND	ND	0.5±0.0	ND	ND	68.0±3.2	115.4±13.6	61.6±79.9

	ChCl/AA	17.3±0.9	8.3±1.1	ND	ND	ND	0.6±0.0	ND	ND	173.0±7.8	36.8±11.2	124.2±5.6
NAT	Ethanol/water 70%	9.8±0.6	11.1±1.1	ND	ND	14.2±0.4	ND	0.6±0.1	ND	34.8±7.8	16.6±8.3	108.5±1.3
	ChCl/urea	30.8±1.2	9.4±0.4	ND	ND	ND	ND	0.3±0.3	ND	17.4±0.3	11.1±4.6	ND
	Bet/LA	25.3±0.5	7.7±0.4	0.3±0.4	ND	ND	ND	0.4±0.0	ND	54.5±2.8	13.6±6.5	ND
	ChCl/AA	19.1±0.0	5.7±0.1	0.5±0.0	ND	ND	ND	ND	ND	94.4±3.5	4.9±0.3	ND
NTC	Ethanol/water 70%	27.7±5.2	31.5±4.4	1.9±0.4	ND	6.1±7.6	0.4±0.6	ND	ND	53.7±17.7	ND	274.0±71.3
	ChCl/urea	43.2±2.7	26.4±1.6	2.1±0.8	ND	ND	ND	ND	0.4±0.0	54.1±3.0	ND	ND
	Bet/LA	30.23±1.4	26.7±2.2	3.4±0.0	ND	ND	0.4±0.0	ND	0.4±0.0	53.1±3.9	ND	ND
	ChCl/AA	21.0±2.4	17.4±1.4	0.8±0.0	ND	ND	1.4±0.1	ND	ND	135.0±2.2	ND	136.2±7.2
NTA	Ethanol/water 70%	13.7±2.1	8.7±1.8	0.8±0.1	ND	ND	0.5±0.0	ND	0.6±0.0	28.0±3.3	ND	240.5±332.8
	ChCl/urea	23.7±3.1	9.7±1.9	1.9±0.7	ND	ND	ND	ND	0.6±0.2	50.8±5.2	ND	ND
	Bet/LA	19.9±4.1	8.7±2.1	2.0±0.3	ND	ND	ND	ND	0.4±0.0	56.4±6.2	ND	ND
	ChCl/AA	12.5±1.5	4.0±0.7	0.7±0.2	ND	ND	0.4±0.1	ND	ND	130.0±3.2	ND	208.2±30.0
MOM	Ethanol/water 70%	13.1±0.4	9.2±0.6	ND	ND	ND	ND	ND	ND	19.3±0.5	ND	125.4±8.9
	ChCl/urea	17.4±0.3	6.8±0.0	ND	ND	ND	ND	ND	ND	26.4±2.9	ND	ND
	Bet/LA	18.4±0.1	7.1±0.2	1.1±0.0	ND	ND	ND	ND	ND	29.1±5.4	ND	ND
	ChCl/AA	15.9±0.9	5.2±0.3	ND	ND	ND	0.2±0.2	ND	ND	145.7±6.0	ND	113.0±1.8
PRB	Ethanol/water 70%	15.1±0.7	24.6±0.1	0.5±0.0	ND	ND	ND	1.2±0.0	0.4±0.0	28.5±0.4	ND	ND
	ChCl/urea	21.7±0.1	19.4±1.5	0.5±0.01	ND	ND	ND	0.9±0.1	0.4±0.0	19.5±0.3	ND	ND
	Bet/LA	17.6±1.8	17.2±1.7	0.7±0.02	ND	ND	ND	0.7±0.1	0.5±0.1	44.9±2.1	ND	ND
	ChCl/AA	14.9±0.8	14.0±0.1	0.4±0.0	ND	ND	0.5±0.0	0.7±0.0	ND	138.9±3.1	ND	ND
PRT	Ethanol/water 70%	13.1±1.0	28.4±0.6	ND	ND	ND	ND	0.7±0.0	ND	30.1±0.8	ND	53.3±68.0
	ChCl/urea	15.5±1.8	16.9±2.3	ND	ND	ND	ND	0.4±0.1	ND	25.6±2.3	ND	ND
	Bet/LA	16.4±0.5	18.6±0.1	0.5±0.0	ND	ND	ND	0.4±0.0	ND	34.5±0.3	ND	ND
	ChCl/AA	15.0±0.1	16.3±0.0	ND	ND	ND	ND	0.4±0.0	ND	107.4±3.0	ND	ND
MTA	Ethanol/water 70%	19.0±0.7	7.6±0.2	ND	48.3±3.3	13.6±0.2	ND	ND	ND	29.6±1.3	ND	417.4±32.0
	ChCl/urea	68.6±28.3	12.0±4.3	ND	68.1±36.8	ND	ND	ND	ND	51.5±17.0	ND	124.4±168.7
	Bet/LA	35.0±1.3	13.5±0.6	2.7±0.0	45.9±3.8	ND	ND	ND	ND	34.1±1.1	ND	ND
	ChCl/AA	31.9v2.3	6.7±0.4	ND	48.7±3.2	ND	1.0±0.0	ND	ND	117.1±0.6	ND	233.4±10.9
MAM	Ethanol/water 70%	7.7±0.6	15.3±0.3	ND	ND	ND	ND	ND	ND	<LOQ	ND	ND
	ChCl/urea	9.0±0.6	7.3±0.6	ND	ND	ND	ND	ND	ND	<LOQ	ND	ND
	Bet/LA	8.7±0.1	6.8±0.3	1.1±0.1	ND	ND	ND	ND	ND	<LOQ	ND	ND
	ChCl/AA	6.9±0.1	2.4±0.2	ND	ND	ND	ND	ND	ND	151.8±6.0	2.9±0.2	ND
NAM	Ethanol/water 70%	5.9±0.3	6.0±1.5	ND	ND	ND	ND	ND	ND	<LOQ	9.0±3.1	55.9±71.8
	ChCl/urea	7.1±0.7	2.3±0.5	ND	ND	ND	ND	ND	ND	<LOQ	<LOQ	ND
	Bet/LA	6.8±0.8	2.0±0.3	ND	ND	ND	ND	ND	ND	<LOQ	<LOQ	ND
	ChCl/AA	9.9±0.4	7.7±0.4	ND	ND	ND	1.0±0.0	ND	ND	120.6±2.6	ND	ND

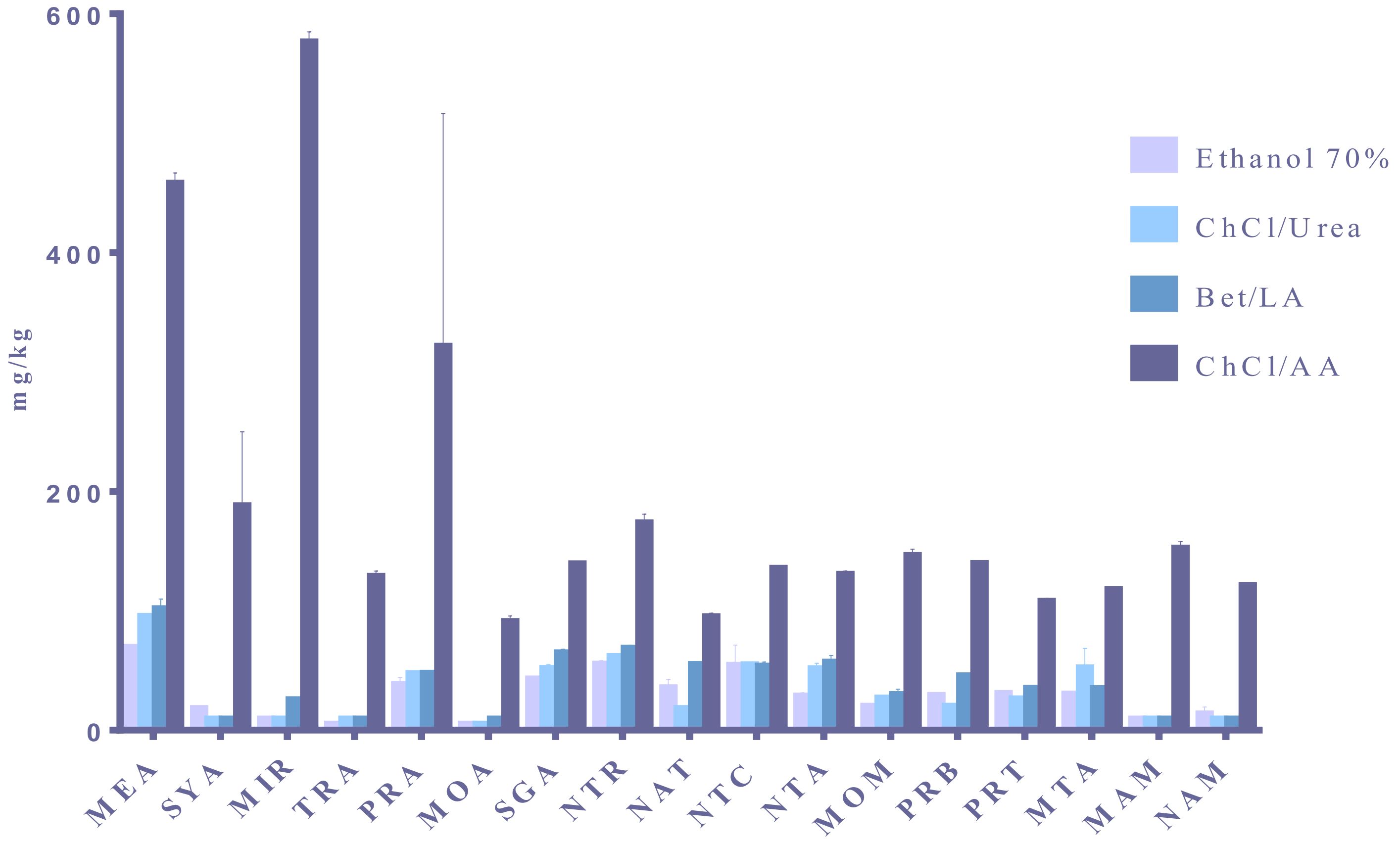

Figure S4. Content of catechin (mg/kg DW) detected in all extracts from dried GPs according to HPLC-DAD analysis.

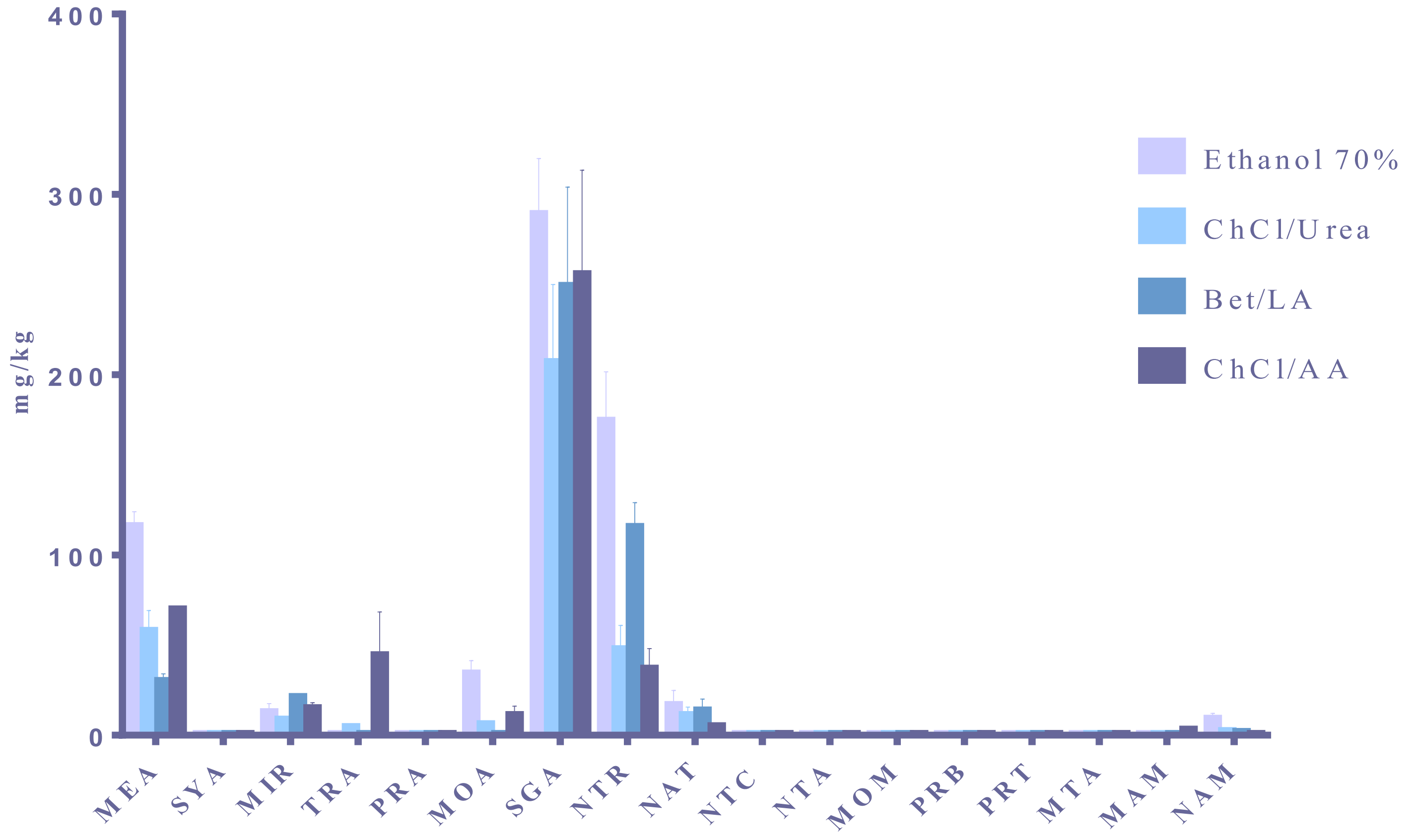

Figure S5. Content of rutin (mg/kg DW) detected in all extracts from dried GPs according to HPLC-DAD analysis.

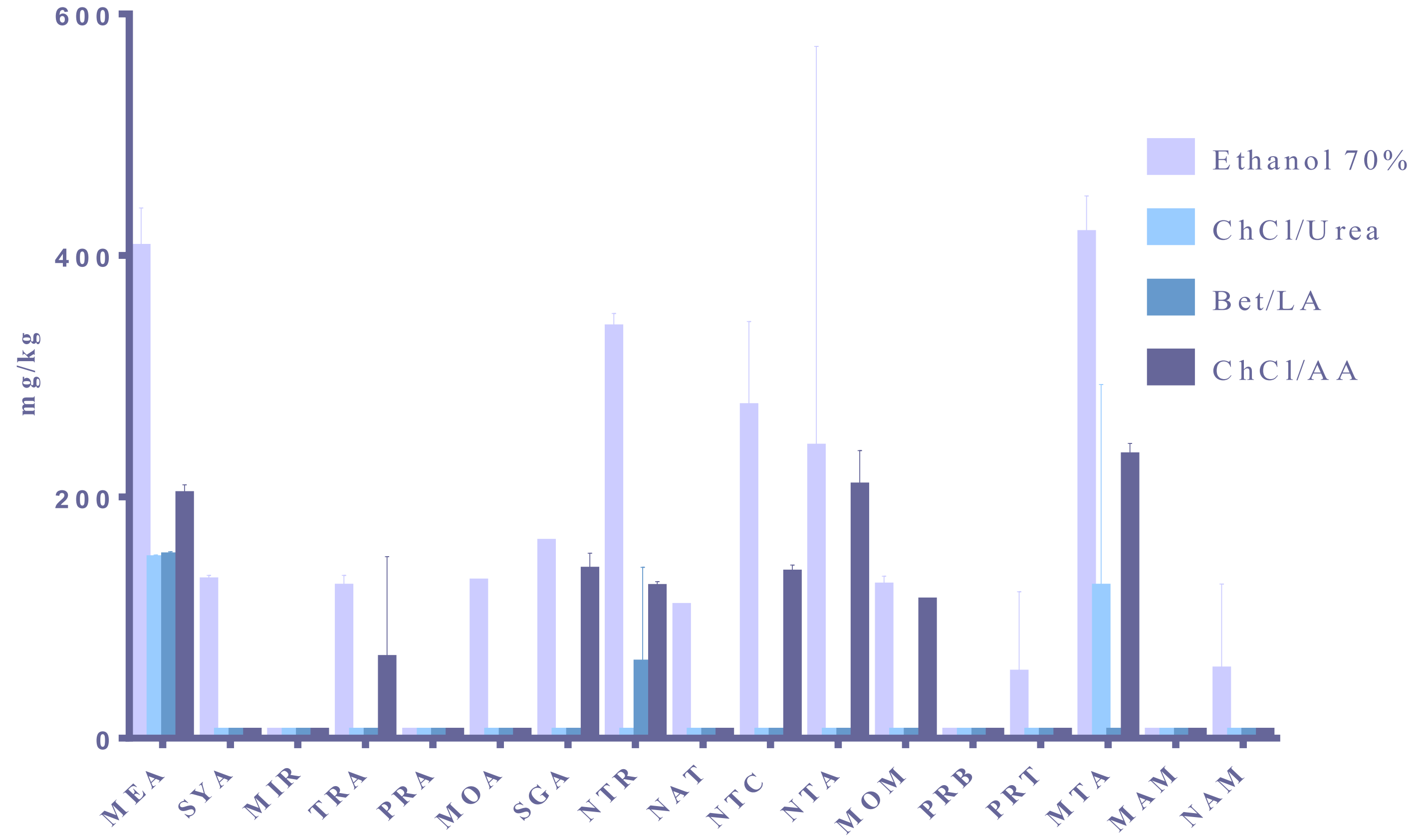

Figure S6. Content of quercetin (mg/kg DW) detected in all extracts from dried GPs according to HPLC-DAD analysis.

Table S3. Phenolic compounds (mg GAE/kg DW) detected in Sangiovese (SGA) and Merlot (MEA) GPs extracted with ethanol 70% (wt/wt), Bet/LA and ChCl/AA NADESs, according to LC-QTOF-MS analysis. ([a])Values as mean ± SD (n = 2).

Phenolic compounds	MEA			SGA		
	Ethanol/water 70%	Bet/LA	ChCl/AA	Ethanol/water 70%	Bet/LA	ChCl/AA
Azelaic acid (mg/kg) [a]	2.5±0.2	0.8±0.2	0.7±0.0	2.1±0.0	ND	ND
Caffeic acid (mg/kg) [a]	3.9±0.5	4.7±0.1	1.2±0.3	0.4±0.2	0.7±0.1	ND
Caffeoyl tartaric acid (mg/kg) [a]	ND	1.9±0.4	ND	ND	2.1±0.4	ND
Chlorogenic acid (mg/kg) [a]	ND	1.7±0.1	ND	ND	ND	ND
Ellagic acid (mg/kg) [a]	2.4±0.3	ND	ND	ND	ND	ND
Fertaric acid (mg/kg) [a]	1.8±0.2	1.2±0.1	0.8±0.3	ND	ND	ND
Feruloyl tartaric acid (mg/kg) [a]	2.2±0.1	3.0±2.5	1.1±0.1	1.6±0.4	4.7±0.8	ND
Gallic acid (mg/kg) [a]	22.8±1.0	ND	ND	18.3±1.8	ND	ND
Gallic acid dihexoside (mg/kg) [a]	ND	ND	ND	ND	6.8±2.5	ND
Gentisic acid (mg/kg) [a]	5.0±0.5	4.1±1.7	1.0±0.0	6.3±0.9	4.7±1.1	0.6±0.1
p-Hydroxybenzoic acid (mg/kg) [a]	11.3±0.1	13.7±5.8	7.6±0.6	27.1±6.2	3.3±0.3	7.0±1.0
Hydroxybenzoic acid hexoside (mg/kg) [a]	ND	ND	ND	0.2±0.0	ND	ND
Dihydroxybenzoic acid hexoside (mg/kg) [a]	ND	ND	ND	1.1±0.1	ND	ND
Ursolic acid (mg/kg) [a]	1.9±0.7	ND	ND	ND	ND	ND
Quinic acid (mg/kg) [a]	ND	1.1±0.3	ND	ND	2.4±0.2	ND
Sinapic acid (mg/kg) [a]	ND	ND	ND	2.1±0.2	ND	ND
Syringic acid (mg/kg) [a]	19.8±0.3	16.4±2.0	7.5±0.3	27.9±6.3	17.2±5.6	7.2±1.4
trans-Cinnamic acid (mg/kg) [a]	3.2±0.3	2.0±0.0	ND	0.8±0.3	ND	ND
Vanillic acid (mg/kg) [a]	1.1±0.1	ND	ND	0.9±0.2	ND	ND
Aromadendrin (mg/kg) [a]	ND	0.5±0.1	ND	1.4±0.4	0.6±0.1	ND

**Aromadendrin-7-O-hexoside (mg/kg)** [a]	1.1±0.1	ND	ND	5.2±2.2	3.5±0.1	ND
**Catechin (mg/kg)** [a]	3.0±0.3	ND	1.7±0.1	1.8±0.2	0.9±0.5	0.8±0.0
**Catechin-3-O-gallate (mg/kg)** [a]	0.6±0.1	ND	ND	ND	ND	ND
**Cyanidin-3-O-arabinoside (mg/kg)** [a]	ND	ND	ND	5.4±0.4	1.7±0.2	ND
**Cyanidin-3-O-glucoside (mg/kg)** [a]	ND	ND	ND	1.8±0.3	ND	ND
**Cyanidin-3-O-sambubioside (mg/kg)** [a]	ND	ND	ND	2.3±0.0	ND	ND
**Galloyl-HHDP-hexoside (mg/kg)** [a]	ND	ND	ND	ND	3.0±0.0	ND
**Delphinidin (mg/kg)** [a]	2.9±0.6	ND	ND	1.2±0.2	ND	ND
**Delphinidin-3,5-di-O-glucoside (mg/kg)** [a]	ND	ND	ND	2.2±0.2	ND	ND
**Delphinidin-3-O-glucoside (mg/kg)** [a]	ND	ND	ND	6.5±1.8	2.0±0.5	ND
**Esculetin (mg/kg)** [a]	4.5±0.1	4.3±0.8	1.7±0.2	3.3±0.1	3.2±0.1	1.1±0.0
**Gingerol (mg/kg)** [a]	1.3±0.2	4.0±2.2	1.0±0.9	1.6±0.6	2.0±0.1	1.2±0.9
**Isorhamnetin (mg/kg)** [a]	ND	ND	ND	6.5±1.1	2.1±0.2	1.0±0.4
**Isorhamnetin-3-O-glucoside (mg/kg)** [a]	ND	2.6±2.7	0.9±0.6	70.1±18.6	21.5±1.0	5.3±6.5
**Isorhamnetin-3-O-glucuronide (mg/kg)** [a]	ND	4.8±0.1	ND	ND	ND	ND
**Kaempferol (mg/kg)** [a]	11.3±1.8	0.8±0.1	ND	4.2±0.0	3.2±0.6	2.1±0.1
**Kaempferol-3-O-glucoside (mg/kg)** [a]	2.9±0.0	1.3±0.2	ND	6.4±0.1	2.9±0.8	1.4±0.5
**Laricitrin (mg/kg)** [a]	ND	0.5±0.1	ND	1.2±0.1	ND	ND
**Laricitrin-3-O-glucoside (mg/kg)** [a]	ND	ND	ND	1.9±0.5	0.9±0.0	0.6±0.1
**Malvidin 3-O-glucoside (mg/kg)** [a]	1.6±0.1	ND	ND	6.7±1.2	ND	1.8±0.0
**Malvidin-3-O-p-coumaroyl glucoside-4-vinylphenol adduct (mg/kg)** [a]	ND	ND	ND	ND	6.8±0.1	ND
**Myricetin (mg/kg)** [a]	1.8±0.2	3.1±0.1	ND	1.3±0.4	1.1±0.1	ND
**Myricetin-3-O-hexoside (mg/kg)** [a]	1.3±0.1	0.8±0.3	ND	3.3±0.4	1.9±0.3	ND
**Naringenin-7-O-glucoside (mg/kg)** [a]	7.0±0.3	2.5±0.1	0.4±0.0	64.8±21.6	19.7±0.7	7.4±3.1

**Tyrosol (mg/kg) [a]**	29.2±1.9	4.3±0.8	ND	32.5±5.1	2.7±0.7	ND
**Pelargonidin-3-O-glucoside (mg/kg) [a]**	1.6±0.2	ND	ND	8.9±1.8	5.0±0.1	2.4±0.2
**Peonidin-3-O-p-coumaroyl glucoside (mg/kg) [a]**	ND	ND	ND	ND	3.2±0.9	ND
**Phloretin (mg/kg) [a]**	1.2±0.0	0.5±0.0	ND	ND	ND	ND
**p-Hydroxybenzaldehyde (mg/kg) [a]**	2.9±0.4	ND	ND	2.2±2.1	2.1±0.1	1.8±0.2
**Piceatannol (mg/kg) [a]**	3.9±2.1	0.8±0.1	1.5±0.1	1.5±0.5	0.7±0.2	0.4±0.0
**Procyanidin dimer B-type (mg/kg) [a]**	3.8±0.2	5.1±0.5	3.6±0.2	2.6±0.3	4.6±0.5	2.9±0.2
**Procyanidin dimer B-type gallate (mg/kg) [a]**	ND	5.9±2.0	ND	ND	ND	1.8±0.1
**Procyanidin dimer B-type digallate (mg/kg)[a]**	ND	0.6±0.0	ND	ND	ND	ND
**Procyanidin trimer B-type (mg/kg) [a]**	ND	3.4±2.4	2.5±0.1	0.9±0.3	5.3±0.2	2.4±0.9
**Quercetin (mg/kg) [a]**	38.3±1.5	2.2±1.9	3.6±0.1	13.5±0.7	0.8±0.2	1.1±0.4
**Quercetin-3,7-di-O-hexoside (mg/kg) [a]**	ND	ND	ND	2.4±0.2	ND	ND
**Quercetin-3-O-glucuronide (mg/kg) [a]**	13.2±0.4	9.6±0.1	2.6±0.4	17.5±4.6	17.3±1.9	4.3±0.6
**Quercetin-3-O-glucoside (mg/kg) [a]**	5.0±0.0	2.1±0.2	ND	9.8±12.4	9.0±1.0	4.2±0.4
**Quercetin 3-O-rutinoside (Rutin) (mg/kg) [a]**	ND	49.2±6.2	ND	21.4±6.6	4.4±0.7	ND
**Resveratrol (mg/kg) [a]**	1.2±0.0	0.5±0.0	ND	ND	ND	ND
**Syringetin-3-O-hexoside (mg/kg) [a]**	2.2±0.4	1.3±0.1	0.8±0.0	3.3±0.1	1.2±0.0	0.8±0.1
**Syringic aldehyde (mg/kg) [a]**	0.8±0.6	0.2±0.3	ND	0.8±0.1	0.2±0.0	ND
